# A Semi-supervised Pipeline for Accurate Neuron Segmentation with Fewer Ground Truth Labels

**DOI:** 10.1523/ENEURO.0352-23.2024

**Published:** 2024-02-09

**Authors:** Casey M. Baker, Yiyang Gong

**Affiliations:** ^1^Departments of Biomedical Engineering, Duke University, Durham, North Carolina 27701; ^2^Neurobiology, Duke University, Durham, North Carolina 27701

**Keywords:** calcium imaging, deep learning, neuron segmentation, semi-supervised

## Abstract

Recent advancements in two-photon calcium imaging have enabled scientists to record the activity of thousands of neurons with cellular resolution. This scope of data collection is crucial to understanding the next generation of neuroscience questions, but analyzing these large recordings requires automated methods for neuron segmentation. Supervised methods for neuron segmentation achieve state of-the-art accuracy and speed but currently require large amounts of manually generated ground truth training labels. We reduced the required number of training labels by designing a semi-supervised pipeline. Our pipeline used neural network ensembling to generate pseudolabels to train a single shallow U-Net. We tested our method on three publicly available datasets and compared our performance to three widely used segmentation methods. Our method outperformed other methods when trained on a small number of ground truth labels and could achieve state-of-the-art accuracy after training on approximately a quarter of the number of ground truth labels as supervised methods. When trained on many ground truth labels, our pipeline attained higher accuracy than that of state-of-the-art methods. Overall, our work will help researchers accurately process large neural recordings while minimizing the time and effort needed to generate manual labels.

## Significance Statement

Modern neuroscience analyzes the activity of hundreds to thousands of neurons from large optical imaging datasets. One important step in this analysis is neuron segmentation. Supervised algorithms have performed neuron segmentation with class-leading accuracy and speed but lag unsupervised algorithms in training time. A large component of training time is the manual labeling of neurons as training samples; current supervised methods train over many manual labels to achieve accurate prediction. We developed a semi-supervised neuron segmentation algorithm, SAND, that retained high accuracy in the few-label regime. SAND employed neural network ensembling to generate robust pseudolabels and used domain-specific hyperparameter optimization. SAND was more accurate than existing supervised and unsupervised algorithms in low and high label regimes of multiple imaging conditions.

## Introduction

Studying modern neuroscience questions requires scientists to simultaneously measure and analyze the coordinated activity of neural ensembles formed from hundreds to thousands of neurons (Stevenson and Kording, 2011; Yuste, 2015; Makino et al., 2017; Stringer et al., 2019; Rumyantsev et al., 2020; Vyas et al., 2020). Understanding the function of neural ensembles is technically challenging because distinctive genetic or functional subtypes of neurons within ensembles spatially overlap and temporally change on timescales ranging from seconds to days (Ziv et al., 2013; Driscoll et al., 2017; Pérez-Ortega et al., 2021; Sweis et al., 2021).

Calcium imaging using fluorescent protein sensors meets these technical recording challenges because it can record neural ensembles with cellular spatial resolution and genetic specificity over multiple months (Nakai et al., 2001; Stosiek et al., 2003; Chen et al., 2013; Y. Zhang et al., 2023). Calcium influx follows action potentials and typically increases the brightness of calcium indicators (Grienberger and Konnerth, 2012). Recent optical setups have successfully recorded the calcium activity of hundreds of thousands of neurons simultaneously (Demas et al., 2021). Modern calcium protein sensors have trended toward detection of single action potentials and linear response over multiple action potentials (Ryan et al., 2023; Y. Zhang et al., 2023).

Cellular or subcellular resolution imaging that captures rapid single-spike calcium transients creates large datasets. Extracting single neuron activity from these large-scale imaging datasets necessitates a pipeline of automated methods; such algorithms could save time and minimize human error during analysis (Stevenson and Kording, 2011). Analysis pipelines usually consist of four steps to predict spiking activity from calcium fluorescence recordings: (1) motion correction, (2) cell segmentation, (3) fluorescence extraction, and (4) spike inference (Theis et al., 2016; Pachitariu et al., 2017; Pnevmatikakis and Giovannucci, 2017; Keemink et al., 2018; Giovannucci et al., 2019; Bao et al., 2022). Automated neuron segmentation in particular has received substantial attention but needs improvement.

Both supervised and unsupervised machine learning methods exist for neuron segmentation (Abbas and Masip, 2022; Bao and Gong, 2023). Supervised methods consist of convolutional neural networks (CNNs), while unsupervised methods include dictionary learning, PCA/ICA, and matrix factorization [e.g., CaImAn (Giovannucci et al., 2019) and Suite2p (Pachitariu et al., 2017)]. Supervised methods are typically more accurate than unsupervised methods (Soltanian-Zadeh et al., 2019; Bao et al., 2021). For example, Shallow U-Net Neuron Segmentation (SUNS) is a supervised deep learning-based pipeline for neuron segmentation that achieves state-of-the-art accuracy and speed (Bao et al., 2021).

Supervised methods trade off superior performance for the large effort required to generate hundreds of ground truth labels for model training and hyperparameter optimization. The many manual labels can help train algorithms that account for idiosyncratic fluorescence and noise distributions within each image dataset but then necessitate labels for each imaging condition. Generating such labels is time consuming and subject to human error (Giovannucci et al., 2019; Zhang et al., 2020).

Semi-supervised learning presents an opportunity to reduce the burdens of manual labeling. Semi-supervised segmentation leverages limited numbers of ground truth labels and unlabeled images to train models using two primary approaches: pseudolabeling and consistency regularization (Ouali et al., 2020). Pseudolabeling increases the size of the training dataset by accepting high-confidence labels predicted on unlabeled data as ground truth labels that can further train the model (Lee, 2013; Zou et al., 2020). Consistency regularization trains models by penalizing dissimilar predictions for similar inputs (Chaitanya et al., 2020; Zhuang et al., 2021; Huang et al., 2022; Wu et al., 2022). A combination of pseudolabeling and consistency regularization significantly improved classification accuracy with small numbers of ground truth labels (Sohn et al., 2020).

An alternative paradigm to semi-supervised learning that improves generalizability is ensemble learning. Ensemble learning improves predictive accuracy by combining the outputs of multiple models (Sagi and Rokach, 2018). Averaging multiple independent models reduces overfitting, increases generalizability, and compensates for high model variability even when trained on limited data (Dietterich, 2002; Polikar, 2006). Previous work has successfully applied ensemble learning to neural networks for image classification and segmentation (Zheng et al., 2019; Muller et al., 2022), with the ensemble outperforming the individual (Krizhevsky et al., 2017).

In this study, we developed a semi-supervised neuron segmentation pipeline that maintained state-of-the-art accuracy and prediction speed while limiting the number of manual training labels. Our approach, Semi-supervised Active Neuron Detection (SAND), used neural network ensemble learning to predict active neurons in unlabeled frames. These predictions acted as pseudolabels to augment our training set. We also developed a novel pipeline to choose algorithm hyperparameters with few ground truth labels.

## Materials and Methods

**Figure 1.**
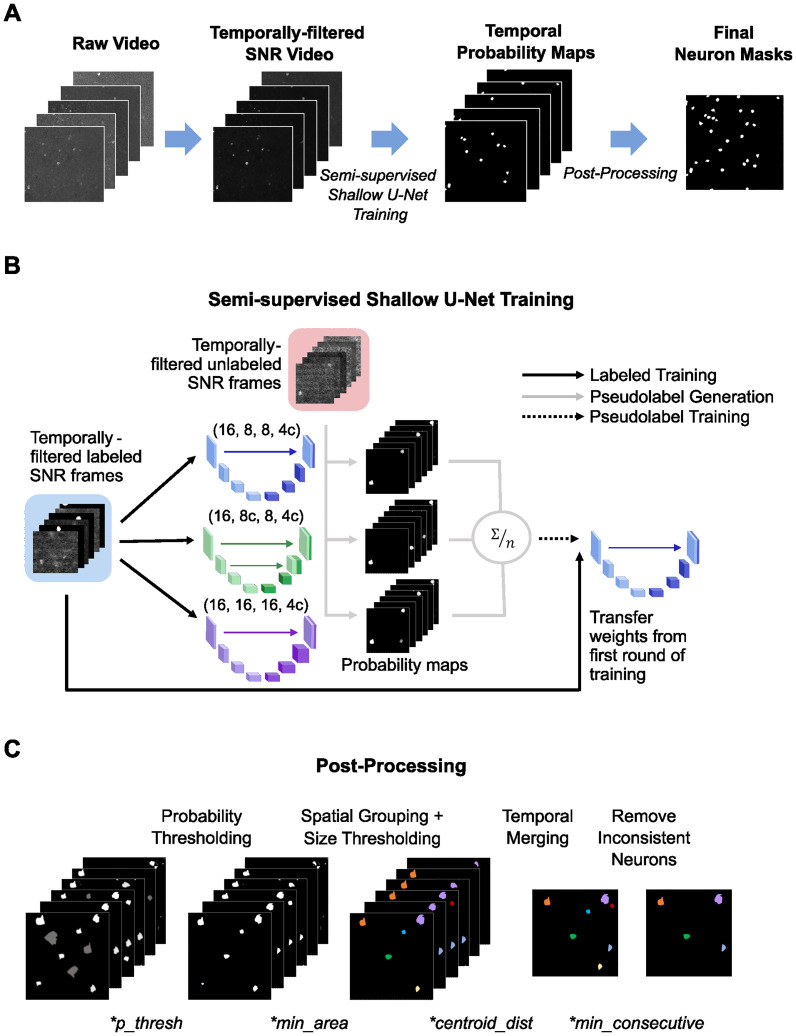
A multistep pipeline processed the input videos into masks using semi-supervised learning and FLHO for postprocessing*. **A***, Examples of preprocessed video frames, intermediate SNR representation frames, model output, and final masks obtained from our pipeline. ***B***, Schematic of our semi-supervised training pipeline that used ensemble learning to predict active neurons in unlabeled frames. We trained three different shallow U-Nets on labeled frames. The titles above the U-Nets represent the number of channels in each level of the decoder, starting with the deepest level, and “c” denotes a concatenation. We then passed unlabeled frames through each model and averaged the resulting probability maps to create pseudolabels. We retrained and fine-tuned one of the three U-Nets with these pseudolabels and then fine-tuned this network with a final round of training on the labeled frames. See Extended Data Figures 1-1, 1-2, 1-3*A*,*B*, and 1-6 for more details. ***C***, FLHO found optimal hyperparameters for the postprocessing pipeline that processed frames of probability maps to predict individual neurons. We first thresholded the probability maps from the CNN (*p_thresh*). We then segmented ROIs in each frame and removed ROIs that were smaller than *min_area*. We then merged ROIs across frames by their relative spatial locations (*centroid_dist*) and removed any ROIs that were not active for enough consecutive frames (*min_consecutive*). * denotes hyperparameters. See Extended Data Figures 1-3*C*, 1-4, and 1-5, Table 1-1, and Table 1-2 for more details.

Our SAND approach consisted of three main steps: (1) preprocessing the entire video to enhance active neurons, (2) semi-supervised CNN training using small numbers of manually labeled frames, and (3) postprocessing to segment unique neuron masks from the CNN output (Fig. 1*A*). The postprocessing step used four hyperparameters. Their values were determined using only the manually labeled frames.

10.1523/ENEURO.0352-23.2024.f1-1 Figure 1-1 **Multiple neural network architectures generated the pseudolabels and final predictions.** We used three U-Net architectures with the same encoder but varying decoders. The labels above the U-Nets represent the number of channels in each level of the decoder, starting with the deepest layer, and ‘c' denotes a concatenation. The numbers above each block represent the number of channels at each layer and the variables to the left represent the dimensions of the image at each step, given an input frame with dimensions *m* × *n*. We used a dropout rate of 0.1 for the first two depths and a rate of 0.2 for the deepest depth. The total number of trained parameters for each architecture was ∼5000 (*blue*), ∼6000 (*green*), and ∼7500 (*purple*). Download Figure 1-1, TIF file.

**Figure 2.**
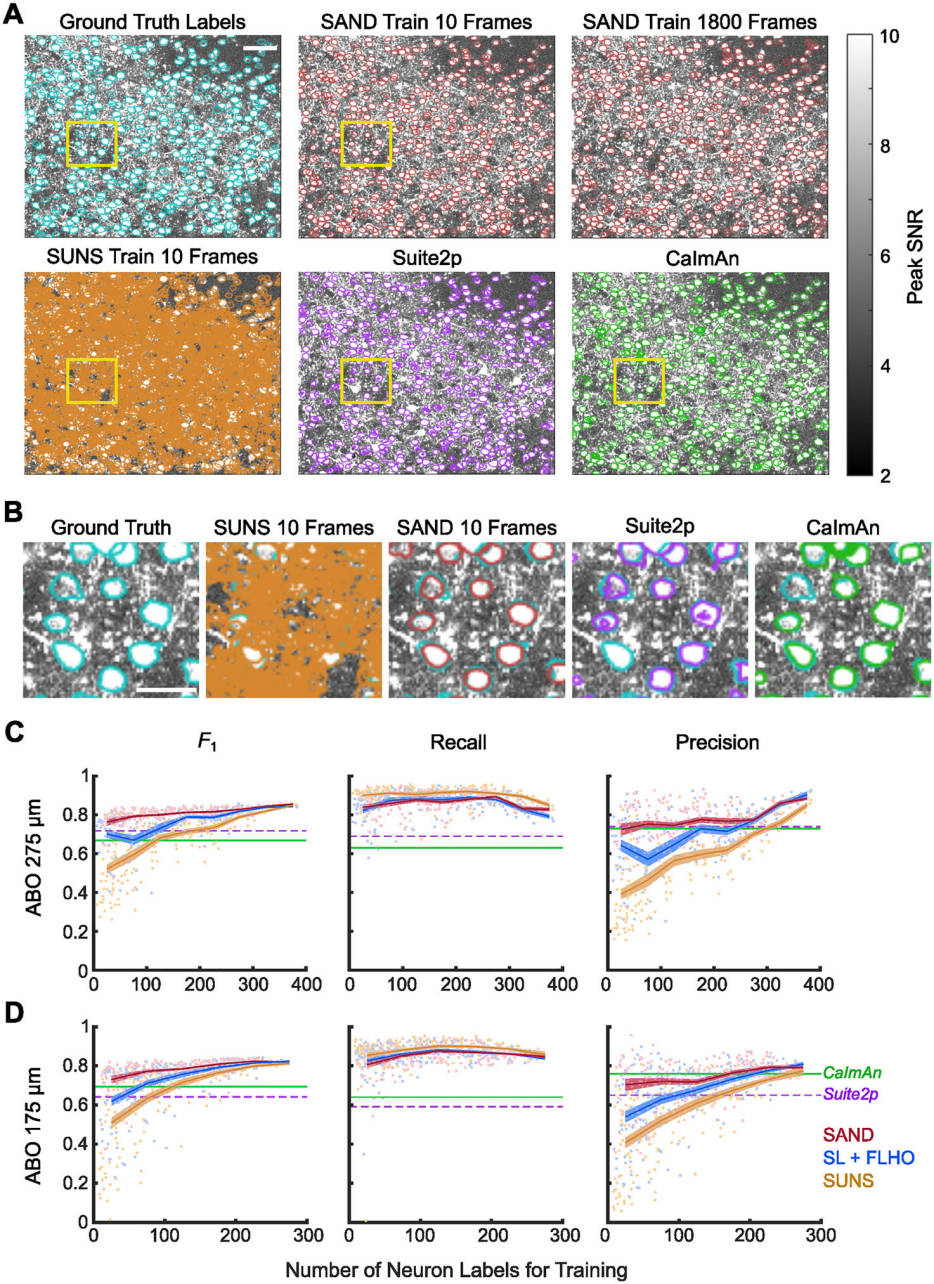
SAND outperformed other pipelines on low numbers of ground truth labels when processing the ABO 275 µm and ABO 175 µm datasets. ***A***, Example segmentations from ABO 275 µm video 539670003. Masks generated by SAND were more accurate than those of other methods, even when trained on only 10 frames. Yellow boxes indicate region isolated in panel ***B***. Scale bar, 50 µm. See Extended Data Figure 2-1*A* and Table 2-2 for more details. ***B***, Example neurons zoomed from boxed regions in panel ***A***. When trained on only 10 frames, SUNS identified many false-positive masks, whereas SAND accurately identified neurons. CaImAn and Suite2p both failed to find some ground truth neurons and Suite2p in particular had irregularly shaped masks. Scale bar, 25 µm. SAND had higher accuracy than other methods with low number of ground truth labels on both the (***C***) ABO 275 µm and (***D***) ABO 175 µm datasets. Dots represent the average *F*_1_ score for each model when processing the nine test videos. Lines represent the mean *F*_1_ scores averaged over bins grouped by the number of training labels; bins spanned 0–50 labels, 50–100 labels, etc. Shaded regions represent standard error. Horizontal lines are the average *F*_1_ scores of Suite2p and CaImAn. SAND generally did not improve recall but improved precision for the ABO datasets. The red line (SAND) represents ensemble learning and hyperparameter optimization with FLHO. The blue line represents single-model supervised learning and hyperparameter optimization with FLHO. The orange line (SUNS) represents single-model supervised learning and grid search hyperparameter optimization. See Extended Data Figures 2-2, 2-3, and 2-4 and Table 2-1 for more details.

10.1523/ENEURO.0352-23.2024.f1-2 Figure 1-2 **Pseudolabel training helped reduce and stabilize training loss. (A**) Example learning curves for training and validation sets using different amounts of training labels on ABO 275 µm data. The first round of supervised training (*green*) lasted for 200 epochs. We then trained models of pseudolabels (*orange*) for 25 epochs. A final round of training on the labeled frames (*purple*) lasted for another 200 epochs. (*Insets*) Corresponding learning curves on pseudolabels, which used binary cross entropy loss. **(B)** Breakdown of training time for SAND and SUNS for each step of training using different amounts of ground truth labels. Total training time for SAND and SUNS increased as the number of training labels increased. Even when trained on 500 frames, both SAND and SUNS could be trained in less than an hour. Bars represent the average training time for 10 different models. Error bars represent the standard deviation for the total training time (*n* = 10 models). Download Figure 1-2, TIF file.

**Figure 3.**
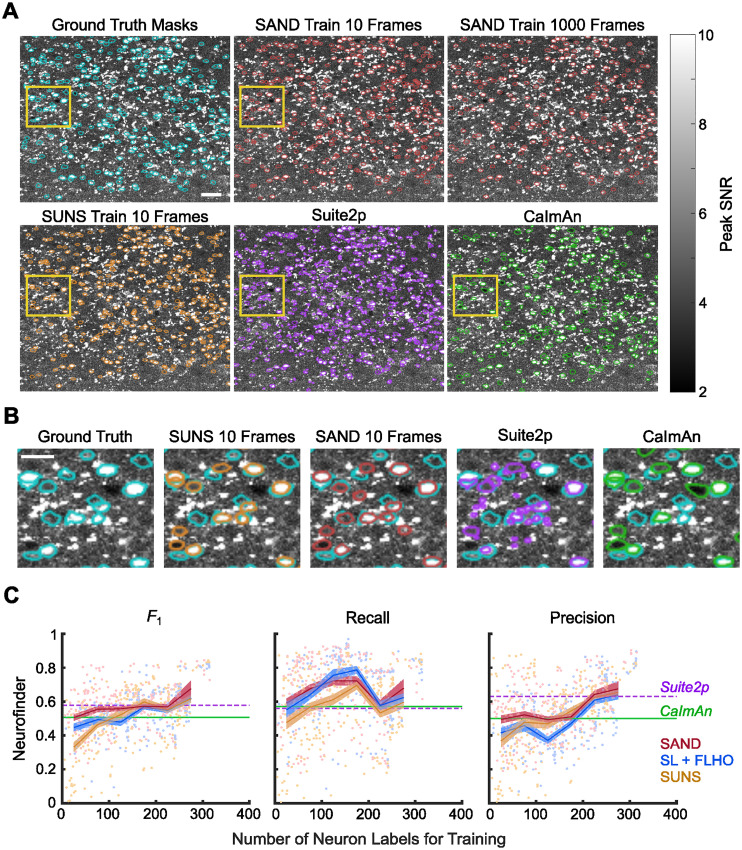
SAND outperformed other pipelines on low numbers of ground truth labels using the Neurofinder datasets. ***A***, Example segmentations from Neurofinder video 4.00. Masks generated by SAND were more accurate than those of other methods, even when trained on only 10 frames. Yellow boxes indicate region isolated in panel ***B***. Scale bar, 50 µm. See Extended Data Figure 2-1*B* and Table 2-3 for more details. ***B***, Example neurons zoomed from boxed regions in panel ***A***. When trained on only 10 frames, SAND correctly identified more masks than CaImAn and Suite2p. Scale bar, 25 µm. ***C***, SAND generally had higher accuracy than other methods when trained on a low number of ground truth labels. Dots represent the average *F*_1_ score for each model when processing the test video(s). Lines represent the mean *F*_1_ scores averaged over bins grouped by the number of training labels; bins spanned 0–50 labels, 50–100 labels, etc. Shaded regions represent standard error. Horizontal lines are the average *F*_1_ scores of Suite2p and CaImAn. More than half of Neurofinder videos did not have >250 neurons, so we did not include trials with >250 neurons in comparisons and binned results. The red line (SAND) represents ensemble learning and hyperparameter optimization with FLHO. The blue line represents single-model supervised learning and hyperparameter optimization with FLHO. The orange line (SUNS) represents single-model supervised learning and grid search hyperparameter optimization. See Extended Data Figure 3-1 and Table 3-1 for more details.

10.1523/ENEURO.0352-23.2024.f1-3 Figure 1-3 **Pseudolabels and final probability maps closely aligned with ground truth temporal masks.** **(A)** Example pseudolabels for two different ABO 275 μm frames generated by ensembles trained on different numbers of labeled frames. Pseudolabels were generally consistent across different numbers of training frames. The scale bar is 50 μm. **(B)** Pseudolabels were strongly correlated with the ground truth temporal masks. Bars represent the median correlation values between 1000 pseudolabels and ground truth temporal masks (100 labels from 10 different models). Error bars represent standard deviation. Data points represent the median correlation values for each model. **(C)** Example probability maps used for FLHO. Probability maps closely aligned with the ground truth temporal mask. Thresholding with the 25^th^ percentile value helped retain lower confidence neurons. The scale bar is 50 μm. Download Figure 1-3, TIF file.

**Figure 4.**
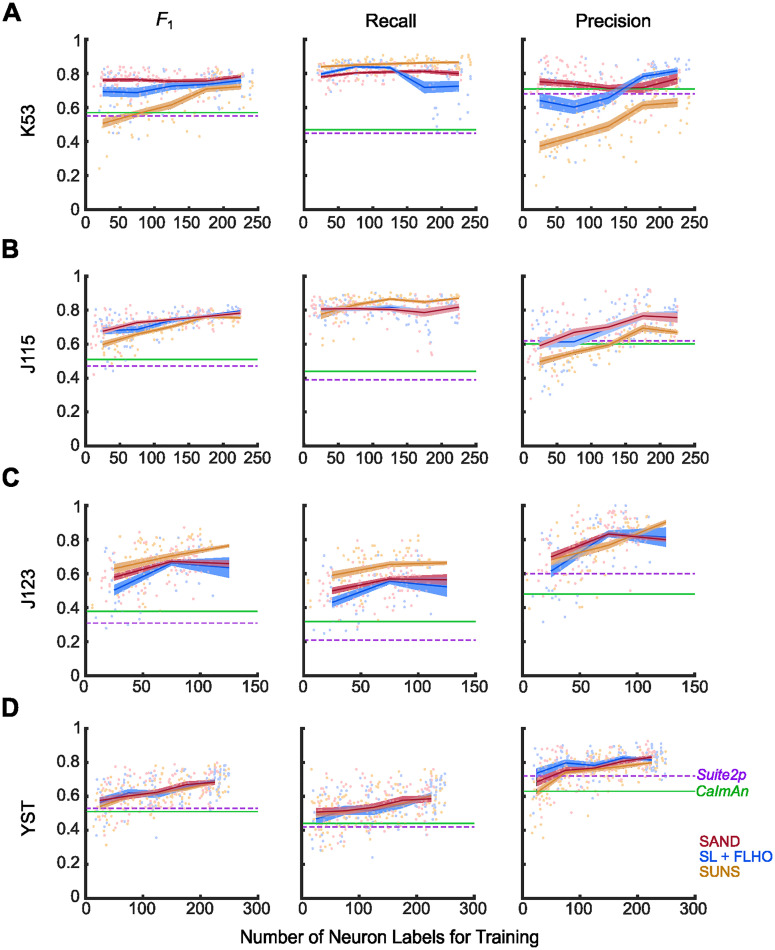
SAND outperformed other pipelines on low numbers of ground truth labels using the CaImAn datasets. ***A***, When trained with low numbers of ground truth neurons, SAND outperformed all other methods on the K53 video. SAND had the highest *F*_1_ and precision of all methods. See Extended Data Figure 2-1*C* and Table 2-4 for more details. ***B***, When trained with low numbers of ground truth neurons, SAND outperformed all other methods on the J115 video. SAND outperformed CaImAn and Suite2p, but not SUNS on the (***C***) J123 and (***D***) YST videos when trained on low numbers of ground truth neurons. Dots represent the average *F*_1_ score for each model when processing the test video(s). Lines represent the mean *F*_1_ scores averaged over bins grouped by the number of training labels; bins spanned 0–50 labels, 50–100 labels, etc. Shaded regions represent standard error. Horizontal lines are the average *F*_1_ scores of Suite2p and CaImAn. The red line (SAND) represents ensemble learning and hyperparameter optimization with FLHO. The blue line represents single-model supervised learning and hyperparameter optimization with FLHO. The orange line (SUNS) represents single-model supervised learning and grid search hyperparameter optimization. See Extended Data Figures 4-1, 4-2, 4-3, 4-4, and 4-5, Table 4-1, and Table 4-2 for more details.

10.1523/ENEURO.0352-23.2024.f1-4 Figure 1-4 **Few label hyperparameter optimization accurately estimated SAND hyperparameters and was invariant to optimization choices. (A)** Grid searches for hyperparameters with small numbers of ground truth labels consistently underestimated the optimal *p_thresh* value. We performed a traditional grid search for all hyperparameters on 20 models, each trained on randomly selected sets of 10 frames from the ABO 275 µm dataset (2 models per video). We also used grid search for all hyperparameters using all labels from all frames to generate the ‘optimal' hyperparameters. *p_thresh* tuned on 10 frames was consistently lower than the optimal *p_thresh*. We then calculated the hyperparameters for the 20 models using our new hyperparameter optimization method, FLHO. This method produced *p_thresh* values that were similar to the optimal *p_thresh*. *p_thresh* is listed as a grayscale pixel value from 0 to 255 (e.g. a value of 205 corresponds to a probability of 80%). Parentheses show the mean ± standard deviation of the number of labeled neurons used in hyperparameter optimization. **(B)** The accuracy of our method was robust to small changes in intermediate *p_thresh*. We performed our hyperparameter optimization pipeline on 20 models using four different percentiles for intermediate *p_thresh*, used during step 2 of the pipeline. Each model was trained on 10 labeled frames from the ABO 275 µm dataset (73 ± 22 labeled neurons). A one-way Kruskal-Wallis test did not find a significant difference in performance between the four choices of *p_thresh* (*p* = 0.20). Bars and error bars respectively represent mean and standard error. **(C)** The values of *centroid_dist* and *min_area* determined by the grid search were robust to changes in the *p_thresh* percentile used in step 2 of FLHO. Bars represent the median values of *centroid_dist* and *min_area* for the models in **B**. A one-way Kruskal-Wallis test did not find a significant difference in the grid search selection between the four choices of *p_thresh* for *min_area* or *centroid_dist* (*n* = 20 models, *p* = 0.45 and *p* = 0.46, respectively). **(D)** The accuracy of our method was robust to changes in the index of *max_continuous* used to calculate *min_consecutive*. We performed our hyperparameter optimization pipeline on 20 models using three different conditions for *min_consecutive* for each model. Each model was trained on 10 labeled frames from the ABO 275 µm dataset (73 ± 22 labeled neurons). A one-way Kruskal-Wallis test did not find a significant difference between the three conditions (*p* = 0.43). Bars and error bars respectively represent mean and standard error. Download Figure 1-4, TIF file.

10.1523/ENEURO.0352-23.2024.f1-5 Figure 1-5 **The Few Label Hyperparameter Optimization pipeline had four main steps.** **(A)** We determined the 25^th^ percentile and median values for *p_thresh*. The 25^th^ percentile value was used in Step 2 and the median value was used in steps 3 and 4, which included neuron prediction. **(B)** We used a grid search to find the *min_area* and *centroid_dist* values that maximized *F*_1_ on labeled frames. **(C)** We found the maximum number of continuous frames for each ground truth neuron. **(D)** We determined the final hyperparameters. We placed an upper bound of 80% on *p_thresh*. To determine *min_consecutive*, we found the second smallest number of continuous frames from Step 3. We placed an upper bound of 8 frames on this value. Download Figure 1-5, TIF file.

10.1523/ENEURO.0352-23.2024.f1-6 Figure 1-6 **We adjusted the number of training labels by randomly sampling different numbers of training frames.** A scatter plot of the number of labeled frames vs. number of labeled neurons for each dataset shows that the number of training labels (neurons) increased as we increased the number of training frames. Each point represents a unique model. Download Figure 1-6, TIF file.

10.1523/ENEURO.0352-23.2024.t1-1 Table 1-1 **The grid search values for post-processing hyperparameters were generally consistent between datasets.** Values are listed as Begin:Step:End. Download Table 1-1, DOCX file.

10.1523/ENEURO.0352-23.2024.t1-2 Table 1-2 **The datasets in this study covered multiple brain regions and imaging conditions.** V1 was primary visual cortex, PPC was posterior parietal cortex, S1 was primary somatosensory cortex, vS1 was vibrissal primary somatosensory cortex. Download Table 1-2, DOCX file.

### Preprocessing

Before training, we preprocessed the video to reduce noise and emphasize active neurons. We first applied pixel-by-pixel temporal filtering to the registered video, which highlighted fluorescence activity that was similar to calcium response waveforms (Bao et al., 2021). We convolved each pixel with the time-reversed average fluorescence response of the ground truth neurons. Selected fluorescence responses had a peak SNR between 5 and 8, and we aligned the transients by their peaks. We then diminished nonresponsive neurons and enhanced active neurons by converting the temporally filtered video into an SNR representation. We calculated this representation by first computing the pixel-wise median image and quantile-based noise image over the entire video. We then pixel-wise subtracted the median image from each frame and pixel-wise divided the result by the noise image.

### Model training

The original SUNS training pipeline used a fully supervised approach and trained a single shallow U-Net with a combination of dice loss and focal loss (Bao et al., 2021). The CNN predicted probability maps that underwent a postprocessing pipeline to calculate the final neuron masks. Our SAND approach used neural network ensembling to generate pseudolabels (Fig. 1*B*). We used an ensemble of three models based on recent work that developed a semi-supervised pipeline for accurate medical image segmentation that trained an ensemble of the same size (Wu et al., 2022). We first defined three separate shallow U-Nets. Each U-Net had a unique decoder architecture, and one U-Net had the same architecture as SUNS (Extended Data Fig. 1-1). We selected the three U-Net architectures tested by Bao et al. (2021) that achieved the highest accuracy. We trained all three U-Nets on frames with manually labeled masks using a weighted sum of dice and focal loss for 200 epochs (focal loss:dice loss, 100:1; Extended Data Fig. 1-2*A*). We then passed 1,800 unlabeled frames through each trained U-Net within the ensemble and averaged the output probability maps to serve as pseudolabels. Pseudolabels closely resembled the known temporal masks (Extended Data Fig. 1-3*A*,*B*). We then produced the final prediction U-Net by using the pseudolabels to continue training the U-Net with the SUNS architecture. We trained this U-Net using binary cross-entropy loss for 25 epochs using the pseudolabels (Extended Data Fig. 1-2*A*), and then we fine-tuned the U-Net with a final round of training using dice and focal loss for 200 epochs using the original labeled frames (Extended Data Fig. 1-2*A*). Training time increased as the number of labeled training frames increased but remained under an hour for up to 500 training frames (Extended Data Fig. 1-2*B*). For all training steps, we used the Adam optimizer with a 0.001 learning rate, and our training pipeline augmented the input frames with random flips and rotations to help prevent overfitting.

### Postprocessing

The output probability maps of our neural network represented the model’s confidence that a pixel belonged to an active neuron. Additional postprocessing converted the output series of probability maps into unique neuron masks (Fig. 1*C*). We followed the same postprocessing steps described in Bao et al. (2021). First, we binarized the probability maps with a probability threshold (*p_thresh*) to determine active pixels. Higher values of *p_thresh* retained only high-confidence predictions. Lower values preserved lower confidence predictions, such as pixels from neurons with relatively low SNR, but also kept more false-positive predictions. After probability thresholding, we grouped active pixels within a frame into separate components using connected component labeling. We removed components smaller than a minimum area (*min_area*), as these regions were unlikely to be neurons. Next, we merged colocalized components across different frames; active components in the same location across multiple frames likely represented the same neuron. We defined components as colocalized if the centers of mass (COMs) of two components were within a minimum threshold (COM distance < *centroid_dist*) or if the areas of two components were substantially overlapping. Overlapped neurons met either of two criteria: (1) intersection-over-union (IoU) > 0.5 or (2) consume ratio (consume) > 0.75, with IoU and consume defined for two binary masks *m*_1_ and *m*_2_ as follows (Bao et al., 2021):

$$\mathrm{IoU}=\frac{\left|m_{1}\cap m_{2}\right|}{\left|m_{1}\cup m_{2}\right|},\;\;$$

$$\mathrm{consume}=\frac{\left|m_{1}\cap m_{2}\right|}{m_{2}}.$$

These temporally merged components represented unique ROIs. Lastly, we removed masks that were not active for a minimum number of consecutive frames (*min_consecutive*) typical of calcium responses.

### Hyperparameter optimization

Selection of the optimal postprocessing hyperparameters after CNN training was crucial for accurately identifying neurons and distinguishing neurons from noise. Hyperparameter optimization with SUNS required manual labeling of all active neurons in the training video. The original SUNS pipeline used a grid search to determine the postprocessing parameters that maximized *F*_1_ on the training frames (Extended Data Table 1-1). Recall, precision, and *F*_1_ are common metrics to define segmentation accuracy:

$$\mathrm{Recall}=\frac{\#\mathrm{True}\;\mathrm{Positives}}{\#\mathrm{Ground}\;\mathrm{Truth}},\;\;$$

$$\mathrm{Precision}=\frac{\#\mathrm{True}\;\mathrm{Positives}}{\#\mathrm{Predicted}},\;\;$$

$$F_{1}\;=\frac{2}{\mathrm{Recal}l^{-1}+\mathrm{Precisio}n^{-1}}.$$

Evaluating *F*_1_ on an entire video is impossible using a video that contains unlabeled neurons, which could be inaccurately labeled as false positives. Similarly, the nature of the *min_consecutive* hyperparameter required all video frames to be used in its estimation. We found that a grid search failed to find the optimal hyperparameters when trained with a small number of labels. In particular, we found that a grid search often underestimated the optimal *p_thresh* value when trained with limited manually labeled frames (Extended Data Fig. 1-4*A*).

We developed a novel pipeline, Few Label Hyperparameter Optimization (FLHO), to optimize postprocessing hyperparameters that used only a fraction of the number of ground truth labels as SUNS (Extended Data Fig. 1-5). Instead of using a grid search to determine all four hyperparameters, we directly calculated *p_thresh* and *min_consecutive* using estimates from a small number of ground truth labels.

We first used the ground truth labels to estimate *p_thresh* (Extended Data Fig. 1-5*A*). For each labeled neuron, we identified the frames when that neuron’s peak SNR (pSNR) exceeded the threshold set by Bao et al. (2021). The trained CNN then calculated probability maps for these active frames. For each neuron, we found the median probability map value within its mask during its active frames. We used this distribution of median probability values for each neuron to find two values: (1) the 25th percentile, which was used for intermediate steps, and (2) the median, which was used as the final *p_thresh*. We used a lower *p_thresh* for intermediate steps that used only labeled frames because our initial small set of labeled training frames likely did not include the frames with the pSNR or peak probability values for each neuron. Our 25th percentile value for *p_thresh* thresholded probability maps and retained neurons with relatively low SNR on the training frames (Extended Data Fig. 1-3*C*). We used these thresholded maps to perform a grid search for values of *centroid_dist* and *min_area* that maximized the *F*_1_ score on the labeled frames (Extended Data Fig. 1-5*B*).

We found that the pipeline was robust across different choice of percentiles with respect to the ultimate algorithm accuracy (Extended Data Fig. 1-4*B*). The values of *centroid_dist* and *min_area* were also robust to changes in *p_thresh*, which may partially explain the robustness in accuracy across percentiles (Extended Data Fig. 1-4*C*). Additionally, the median value of the *p_thresh* distribution trained on a small number of labels was very similar to the optimal *p_thresh* value calculated using all labels (Extended Data Fig. 1-4*A*). Therefore, we set our final *p_thresh* to the median value. We set an upper bound on this value so that *p_thresh* was not >80% probability. Finally, we calculated *min_consecutive* by assessing the distribution of consecutive frames for all neurons (Extended Data Fig. 1-5*C*). For this step, we used the probability maps for all frames. Therefore, we set *p_thresh* to its final (median) value. We thresholded these probability maps using *p_thresh* and *min_area*. We calculated the maximum number of consecutive frames that the model identified for each neuron. We observed that the minimum consecutive frame value among all neurons was occasionally an outlier, so we selected the second smallest value to be *min_consecutive*. However, the performance of our method was robust across different choices of *min_consecutive* (Extended Data Fig. 1-4*D*). We set an upper bound on *min_consecutive* so that it did not surpass eight frames (Extended Data Fig. 1-5*D*).

### Peer segmentation methods

#### SUNS

SUNS is a supervised deep learning pipeline for neuron segmentation from fluorescence recordings (Bao et al., 2021). SUNS first computed an SNR representation of imaging videos that emphasized active neurons and de-emphasized inactive neurons. SUNS then trained a shallow U-Net on 1,800–2,400 of all imaging frames developed from a set of comprehensively labeled neurons over all imaging movies. Finally, a multistep postprocessing pipeline identified unique ROIs across all frames. SUNS determined the hyperparameters for this postprocessing pipeline with a grid search that evaluated accuracy against the ground truth labels. Python code for SUNS is available at https://github.com/YijunBao/Shallow-UNet-Neuron-Segmentation_SUNS.

#### CaImAn

CaImAn is a calcium imaging analysis pipeline that uses both unsupervised and supervised algorithms to identify active neurons (Pnevmatikakis et al., 2016; Giovannucci et al., 2019). The unsupervised step was a nonnegative matrix factorization method that separated spatially overlapping neurons based on the temporal activity of active neurons; these sparse decomposed components also included sources that represented background noise and neuropil activity. Components representing unique regions of interest (ROIs) were curated by iteratively combining components that exceeded a threshold for correlated temporal activity. The supervised portion was a quality control step to remove non-neuronal components. This step used a peak signal-to-noise (SNR) threshold, spatial footprint consistency, and a CNN classifier. Python code for CaImAn is available at https://github.com/flatironinstitute/CaImAn (version 1.6.4).

#### Suite2p

Suite2p is another widely used pipeline that applies unsupervised algorithms to identify potential neurons and a supervised quality control step to refine the neurons (Pachitariu et al., 2017). Suite2p first reduced the dimensionality of the input video using singular value decomposition. Then, unsupervised non-negative matrix factorization identified ROIs and modeled decomposed neural activity as the weighted sum of underlying neural activity and neuropil signal. A supervised classifier then processed these ROIs and separated cells from noncells based on temporal and spatial features. Lastly, manual acceptance or rejection of the classifier’s predictions refined the final output neurons. Python code for Suite2p is available at https://github.com/MouseLand/suite2p (version 0.6.16).

### Datasets

We tested our pipeline on two-photon videos from three different datasets, all recorded in mice. These videos covered multiple cortical and subcortical brain regions, were collected with multiple imaging conditions, and utilized various calcium sensors with different responses and kinetics (Extended Data Table 1-2).

#### Allen Brain Observatory

The dataset from the Allen Brain Observatory (ABO) consisted of 10 videos recorded from a depth of 275 µm and 10 videos recorded from a depth of 175 µm in the primary visual cortex (V1; de Vries et al., 2020). The 175 µm set had ∼200 neurons per video, and the 275 µm set had ∼300 neurons per video. For each depth, we used 10-fold cross-validation: we trained our model and determined the hyperparameters using one video and tested on the other nine videos. Data is available at http://observatory.brain-map.org/visualcoding*.*

#### Neurofinder

We used three sets of videos (01, 02, and 04) from three different labs with different imaging conditions from the Neurofinder competition (CodeNeuro, 2016). Each video was paired with another video obtained under the same imaging conditions, making six pairs of videos. For each of the six pairs, we trained the model and determined the hyperparameters on one video and tested on the other video. The 12 videos averaged ∼250 neurons per video. Videos are available at http://Neurofinder.Codeneuro.Org/.

#### CaImAn

The CaImAn dataset (Giovannucci et al., 2019) contained four videos (J115, J123, K53, and YST) that imaged various brain regions. We divided each video into quarters to perform cross-validation, so that the training and test set had the same imaging conditions. For two of the videos (J115 and K53), the average number of neurons per subvideo was ∼200. For these videos, we trained the model on one subvideo and tested on the remaining three subvideos. The other two videos (J123 and YST) had ∼40 and ∼80 neurons per subvideo, respectively. For these videos containing far fewer neurons, we used leave-one-out cross-validation, training on three subvideos, and testing on the remaining subvideo.

### Analysis

We compared three different deep learning segmentation pipelines: (1) SUNS, model training with supervised learning (SL) and hyperparameter optimization with a full grid search (GS), (2) SL and our new hyperparameter optimization pipeline (FLHO), and (3) SAND, model training using a combination of SL and neural ensemble learning and hyperparameter optimization with FLHO. We also compared SAND with the widely used matrix factorization methods Suite2p and CaImAn. We quantified the quality of the identified masks as the proportion of the mask’s area divided by the area of the mask’s convex hull. We evaluated model accuracy by calculating the *F*_1_ score of each method on the test videos when trained with different numbers of ground truth neuron masks from the training video. We altered the number of ground truth masks used in training by randomly sampling different sets of SNR frames (Extended Data Fig. 1-6). We evaluated *F*_1_ across all frames and neurons in the test videos using the same ground truth masks as previous work (Soltanian-Zadeh et al., 2019; Bao et al., 2021). For CaImAn and Suite2p, we used the *F*_1_ values found in Bao et al. (2021), which previously optimized the hyperparameters for these pipelines.

We ran multiple analyses to test the performance of SAND. First, we compared SAND with SUNS, SL + FLHO, CaImAn, and Suite2p when trained on a low number of ground truth neurons. We also compared the performance of SAND trained on a low number of ground truth neurons with the asymptotic performance of SUNS. Finally, we compared the asymptotic performance of SAND with the asymptotic performance of SUNS. We binned the *F*_1_ scores for each condition by the number of neurons used in training. We compared algorithms using the Wilcoxon rank-sum test and by computing the effect size (Cohen’s *d*).

### Code accessibility

The code described in the paper is available at https://github.com/caseymbaker/SemiSupervisedNeuronSegmentation2p. The GitHub repository includes a tutorial for downloading and running SAND as well as the data and code for recreating figures in this paper. Code was tested on two Windows 10 PCs (AMD Ryzen 9 3900X, 128 GB RAM, RTX 2080, and Intel Core i7-7700K, 64 GB RAM, Quadro P5000).

10.1523/ENEURO.0352-23.2024.suppl-1 Supplementary 1 Download Suppl 1, ZIP file.

## Results

We first evaluated SAND using both ABO datasets (Fig. 2). Masks generated by SAND closely matched the ground truth masks even when trained on only 10 frames (Fig. 2*A*,*B*). Masks generated by SUNS trained on few frames and, however, included many false positives, and masks generated by Suite2p and CaImAn were more irregularly shaped and less accurate than those generated by SAND (Fig. 2*A*,*B*; Extended Data Fig. 2-1*A*; Extended Data Tables 2-1, 2-2). SAND significantly outperformed all other methods when trained on 0–50 ground truth labels (∼10 labeled frames; Extended Data Fig. 2-2, Table 2-1). In the 275 µm dataset, SUNS achieved a median *F*_1_ score of 0.81 when trained on >250 labels (Fig. 2*C*; Extended Data Table 2-1). However, SAND achieved this *F*_1_ score when trained on only ∼25% the number of labels and came within one standard deviation of this value when trained on only ∼12% the number of labels (median *F*_1_ = 0.79; 34 ± 10 neurons). Additionally, the *F*_1_ score for SAND when trained on >250 neurons was significantly higher than the SUNS *F*_1_ score (Extended Data Table 2-1). In the 175 µm dataset (Fig. 2*D*), SUNS achieved a median *F*_1_ score of 0.81 when trained on >200 neuron labels (Extended Data Table 2-1). However, SAND came within one standard deviation of this value when trained on only ∼13% of the number of labels (median *F*_1_ = 0.77, 29 ± 12 neurons). Additionally, the *F*_1_ score for SAND when trained on >200 labels was significantly higher than the SUNS *F*_1_ score when trained on >200 labels (Extended Data Table 2-1). SAND also significantly outperformed the matrix factorization methods, CaImAn and Suite2p, over all numbers of ground truth masks (Extended Data Table 2-1). In particular, SAND trained on only 10 frames more reliably detected low pSNR neurons than CaImAn and Suite2p (Extended Data Fig. 2-3). SAND generally improved model precision (Fig. 2*C*,*D*). Both our new training method and our new hyperparameter optimization method helped maximize *F*_1_ in our pipeline. FLHO without pseudolabel training (SL + FLHO) had a modest effect on accuracy when trained on fewer ground truth masks (Fig. *2C*,*D*). In addition to state-of-the-art accuracy, SAND also achieved the state-of-the-art processing speed of SUNS at ∼300 frames per second (Extended Data Fig. 2-4).

10.1523/ENEURO.0352-23.2024.f2-1 Figure 2-1 **SAND had higher quality masks than competing methods. (A)** Masks identified by SAND were more consistently shaped like neuron somas than masks identified by other methods on the ABO datasets. SUNS and SAND were both trained on 10 frames. **(B)** Masks identified by SAND were more consistently shaped like neuron somas than masks identified by other methods on the Neurofinder dataset. SUNS and SAND were both trained on 10 frames. **(C)** Masks identified by SAND were more consistently shaped like neuron somas than masks identified by other methods on the CaImAn datasets. SUNS and SAND were both trained on 10 frames for J115, K53, and YST. SUNS and SAND were both trained on 100 frames for J123. Bars represent average ratio values and error bars represent standard deviation. *** indicates *p* < 0.001 (**Tables 2-2, 2-3, 2-4**). Download Figure 2-1, TIF file.

10.1523/ENEURO.0352-23.2024.f2-2 Figure 2-2 **SAND outperforms other methods on the ABO dataset when trained on fewer labeled frames.** SAND had higher accuracy than other methods with low number of labeled frames on both the ABO 275 μm and ABO 175 μm datasets. Dots represent the average *F*_1_ score for each model when processing the nine test videos. Lines represent the mean *F*_1_ scores averaged over different numbers of training frames. Shaded regions represent standard error. Horizontal lines are the average *F*_1_ scores of Suite2p and CaImAn. Download Figure 2-2, TIF file.

10.1523/ENEURO.0352-23.2024.f2-3 Figure 2-3 **Neuron recall with SAND outperformed unsupervised algorithms.** We calculated recall as a function of neuron pSNR for each method on the ABO 275 μm dataset. SAND trained on 10 frames could more reliability detect neurons, specifically in the low pSNR regime, compared to CaImAn and Suite2p. SUNS trained on 10 frames had higher recall than SAND in the low pSNR regime, but also had lower precision than all other methods (**Figure 2C**). Lines represent the average recall across all 10 models for all 10 videos. Neurons were grouped by their pSNR in bins with a width of 3 (*n* = 3016 neurons). Download Figure 2-3, TIF file.

10.1523/ENEURO.0352-23.2024.f2-4 Figure 2-4 **SAND achieved the same processing speed as SUNS on the ABO 275 μm dataset.** Segmentation with SAND and SUNS consisted of three steps: pre-processing, CNN inference, and post-processing. For all steps, SAND and SUNS achieved comparable speeds. The total processing speed was an order of magnitude faster than the video’s frame rate (30 Hz). Bars represent the average processing speeds for 10 different models over 9 videos. Error bars represent the standard deviation. Download Figure 2-4, TIF file.

10.1523/ENEURO.0352-23.2024.t2-1 Table 2-1 **SAND significantly outperformed SUNS on the ABO 275 µm and ABO 175 µm datasets when both trained on a small number of labels.** SAND also significantly outperformed SUNS when trained on many labels. "Neuron #" indicates the range of labeled neurons used to train our models over many trials, grouped as a bin; the average number of training labels for each of these trials followed in parentheses. "Neuron #" for CaImAn and Suite2p is listed as NA because these methods were unsupervised; however, hyperparameter optimization for these unsupervised methods was done using all ground truth labels. *n*_*x*_ shows the number of trials (models) in that bin. *F*_1_ is the median *F*_1_ value in that bin. We used a two-sided Wilcoxon rank-sum test to evaluate significance. *, **, ***, and n.s. represent *p* < 0.05, 0.01, 0.001, and not significant, respectively. Download Table 2-1, DOCX file.

10.1523/ENEURO.0352-23.2024.t2-2 Table 2-2 **SAND had significantly higher quality masks than competing methods on the ABO datasets.** We measured quality as the ratio of the mask’s area to the area of the mask's convex hull. SAND and SUNS used 10-fold cross validation to test performance; each model trained on a single video and was tested on the 9 remaining videos in that dataset (i.e. 9 applied models per video). CaImAn and Suite2p did not use cross validation (1 applied model per video). We evaluated SAND and SUNS based on models trained on 10 labeled frames. "# Training Frames" for Suite2p and CaImAn are N/A because these methods were unsupervised. We compared methods using a two-tailed Wilcoxon rank-sum test on all the masks generated by each model across all test videos. Download Table 2-2, DOCX file.

10.1523/ENEURO.0352-23.2024.t2-3 Table 2-3 **SAND had significantly higher quality masks than competing methods on the Neurofinder dataset.** We measured quality as the ratio of the mask's area to the area of the mask’s convex hull. SAND and SUNS used Train 1 Test 1 cross validation to test performance; each model was trained on 1 video and tested on a corresponding video (i.e. 1 applied model per video). CaImAn and Suite2p did not use cross validation (1 applied model per video). We evaluated SAND and SUNS based on models trained on 10 labeled frames. "# Training Frames" for Suite2p and CaImAn are N/A because these methods were unsupervised. We compared methods using a two-tailed Wilcoxon rank-sum test on all the masks generated by each model across all test videos. Download Table 2-3, DOCX file.

10.1523/ENEURO.0352-23.2024.t2-4 Table 2-4 **SAND had significantly higher quality masks than competing methods on the CaImAn datasets.** We measured quality as the ratio of the mask's area to the area of the mask's convex hull. SAND and SUNS trained on 1 video for the K53 and J115 datasets and tested on the remaining 3 videos (i.e. 3 applied models per video). SAND and SUNS trained on 3 videos for the J123 and YST datasets and tested on the held-out video (1 applied model per video). "# Training Frames" for Suite2p and CaImAn are N/A because these methods were unsupervised. We compared methods using a two-tailed Wilcoxon rank-sum test on all the masks generated by each model across all test videos. Download Table 2-4, DOCX file.

We next tested SAND on the Neurofinder dataset (Fig. 3). Masks generated by SAND closely matched the ground truth masks even when trained on only 10 frames (Fig. 3*A*,*B*). Masks generated by Suite2p and CaImAn were more irregularly shaped and had more false-negative predictions than SAND (Fig. 3*A*,*B*; Extended Data Fig. 2-1*B*, Table 2-3). SAND significantly outperformed SUNS when trained on 0–50 ground truth neuron labels (∼10 frames; Fig. 3*C*; Extended Data Fig. 3-1, Table 3-1). SUNS achieved a median *F*_1_ score of 0.58 when trained on 200–250 labels. However, the performance of SAND was not significantly different from this when trained on only ∼14% the number of labels (median *F*_1_ = 0.53, 32 ± 12 neurons; Extended Data Table 3-1). Similar to observations when processing the ABO datasets, our new hyperparameter optimization without pseudolabel training partially improved accuracy when trained on fewer ground truth masks. SAND performed as well as or better than CaImAn segmentation over all numbers of ground truth masks (Extended Data Table 3-1). Overall, the Neurofinder dataset had the most variability in performance, likely due to the variety of imaging conditions throughout this dataset.

10.1523/ENEURO.0352-23.2024.f3-1 Figure 3-1 **SAND outperforms SUNS on the Neurofinder dataset when trained on fewer labeled frames.** SAND had higher precision and recall than SUNS when trained on only 10 labeled frames. Dots represent the average *F*_1_ score for each model when processing the nine test videos. Lines represent the mean *F*_1_ scores averaged over different numbers of training frames. Shaded regions represent standard error. Horizontal lines are the average *F*_1_ scores of Suite2p and CaImAn. Download Figure 3-1, TIF file.

10.1523/ENEURO.0352-23.2024.t3-1 Table 3-1 **SAND significantly outperformed SUNS on the Neurofinder dataset when both trained on a small number of labels.** SAND trained on 0-50 labels did not significantly differ from SUNS trained on 200-250 labels. "Neuron #" indicates the range of labeled neurons used to train our models over many trials, grouped as a bin; the average number of training labels for each of these trials followed in parentheses. "Neuron #" for CaImAn and Suite2p is listed as NA because these methods were unsupervised; however, hyperparameter optimization for these unsupervised methods was done using all ground truth labels. *n_x_* shows the number of trials (models) in that bin. *F*_1_ is the median *F*_1_ value in that bin. We used a two-sided Wilcoxon rank-sum test to evaluate significance. *, **, ***, and n.s. represent *p* < 0.05, 0.01, 0.001, and not significant, respectively. Download Table 3-1, DOCX file.

Finally, we tested SAND on the CaImAn dataset, starting with the K53 and J115 videos (Fig. 4*A*,*B*). When processing the K53 dataset, SAND significantly outperformed SUNS, Suite2p, and CaImAn at all numbers of ground truth neurons (Extended Data Table 4-1). SAND’s performance when trained on 0–50 neurons (∼10–25 frames) was more accurate than the performance of SUNS using >150 ground truth neurons (∼500–1,800 frames; Fig. 4*A*; Extended Data Fig. 4-1, Table 4-1). When processing the J115 dataset, SAND significantly outperformed CaImAn and Suite2p on all numbers of ground truth neurons (Fig. 4*B*; Extended Data Table 4-1). SAND also significantly outperformed SUNS when trained on 0–50 ground truth neuron labels (∼10 frames; Extended Data Fig. 4-1, Table 4-1). For both videos, SAND’s predicted masks aligned closely with the ground truth masks, even when trained on just 10 frames (Extended Data Fig. 4-2). SUNS’s predicted masks included many false positives. Conversely, CaImAn and Suite2p both failed to detect many ground truth neurons. SAND outperformed CaImAn and Suite2p on both the YST and J123 videos on all numbers of ground truth neurons; however, SAND did not consistently outperform SUNS (Fig. 4*C*,*D*; Extended Data Fig. 4-3). On all of the CaImAn videos, SAND predicted masks with more consistent soma shapes than other methods (Extended Data Fig. 2-1C, Table 2-4).

10.1523/ENEURO.0352-23.2024.f4-1 Figure 4-1 **SAND outperforms SUNS on K53 and J115 when trained on fewer labeled frames.** SAND had higher precision than SUNS when trained on only 10 labeled frames from the K53 and J115 videos. Dots represent the average *F*_1_ score for each model when processing the nine test videos. Lines represent the mean *F*_1_ scores averaged over different numbers of training frames. Shaded regions represent standard error. Horizontal lines are the average *F*_1_ scores of Suite2p and CaImAn. Download Figure 4-1, TIF file.

10.1523/ENEURO.0352-23.2024.f4-2 Figure 4-2 **Masks generated by SAND closely matched ground truth masks on the K53 and J115 videos.** **(A)** Example segmentations from a K53 sub-video. Masks generated by SAND were more accurate than those of other methods, even when trained on only 10 frames. The scale bar is 10 μm. **(B)** Example segmentations from a J115 sub-video. Masks generated by SAND were more accurate than those of other methods, even when trained on only 10 frames. For both videos, SUNS’s predictions included many false positives, while CaImAn and Suite2p had many false negatives. The scale bar is 10 μm. Download Figure 4-2, TIF file.

10.1523/ENEURO.0352-23.2024.f4-3 Figure 4-3 **SAND and SUNS predicted similar neuron masks from the J123 and YST videos. (A)** Example segmentations from a J123 sub-video. The scale bar is 25 μm. **(B)** Example segmentations from a YST sub-video. The scale bar is 10 μm. Download Figure 4-3, TIF file.

10.1523/ENEURO.0352-23.2024.f4-4 Figure 4-4 **SAND greatly outperformed SUNS on datasets with high average pSNR or low variability of pSNR.** Scatter plot of average vs standard error of pSNR for all neurons in each video. Dot size indicates the difference in median *F*_1_ between SAND and SUNS when trained on 0-50 neurons. Green dots indicate datasets where SAND significantly outperformed SUNS on low numbers of ground truth labels. Download Figure 4-4, TIF file.

10.1523/ENEURO.0352-23.2024.f4-5 Figure 4-5 **SAND generalized well to videos with similar imaging conditions as the training data.** We trained SAND (*red*) and SUNS (*orange*) on the ABO 175 μm dataset and tested the performance of those models on a dataset with similar imaging conditions (ABO 275 μm) and a dataset with different imaging conditions (K53). We evaluated these models against CaImAn (*green*) and Suite2p (*purple*) using the optimal hyperparameters for each test dataset. We also evaluated the models against SAND models that were trained on videos with the same imaging conditions as the test data (*gray*). SAND generalized well to datasets with similar imaging conditions to the training data. SAND outperformed unsupervised methods when generalizing to data with different imaging conditions. However, SAND performed best when training and testing videos had the same imaging conditions. Download Figure 4-5, TIF file.

10.1523/ENEURO.0352-23.2024.t4-1 Table 4-1 **SAND significantly outperformed SUNS on the K53 and J115 videos when both trained on a small number of labels.** SAND also significantly outperformed SUNS when trained on many labels. "Neuron #" indicates the range of labeled neurons used to train our models over many trials, grouped as a bin; the average number of training labels for each of these trials followed in parentheses. "Neuron #" for CaImAn and Suite2p is listed as NA because these methods were unsupervised; however, hyperparameter optimization for these unsupervised methods was done using all ground truth labels. *n_x_* shows the number of trials (models) in that bin. *F*_1_ is the median *F*_1_ value in that bin. We used a two-sided Wilcoxon rank-sum test to evaluate significance. *, **, ***, and n.s. represent *p* < 0.05, 0.01, 0.001, and not significant, respectively. Download Table 4-1, DOCX file.

10.1523/ENEURO.0352-23.2024.t4-2 Table 4-2 **Calcium sensor SNR have improved over multiple generations of development.** SNR values are the average fold increase of SNR relative to GCaMP3. Values are estimated from (Y. Zhang et al., 2023) and (Chen et al., 2013) and are based on single action potential results *in vitro*. Download Table 4-2, DOCX file.

To understand why SAND only moderately outperformed SUNS when processing the J123 and YST videos, we compared the quality of these videos to the quality of the other datasets. The pSNR of a neuron’s fluorescence can predict likeliness of being detected by both supervised and unsupervised segmentation methods: neurons with higher pSNR were more likely to be detected (Bao et al., 2021). We calculated the average and standard error of pSNR for all ground truth neurons in each video (Extended Data Fig. 4-4).

Neurons in J123 and YST had both lower average pSNR and more variable pSNR than neurons in other videos. This suggests that SAND works best on videos with high pSNR values and low variability of pSNR across neurons. However, SAND appears to be effective when only one of these conditions is met. For example, SAND effectively processed video K53, which had high pSNR but high variability; it also effectively processed the Neurofinder dataset, which had low variability but low pSNR.

The type of calcium indicator used in each recording impacted the pSNR values. Notably, the J123 and YST videos used GCaMP5 (Akerboom et al., 2012) and GCaMP3 (Tian et al., 2009), respectively. These older sensors have very low SNR relative to modern sensors, such as the GCaMP6 used in the other videos (Extended Data Table 4-2). Protein sensors of calcium have continued to develop, so recent sensors in the GCaMP8 series have even higher SNR than that of GCaMP6 (Chen et al., 2013; Ryan et al., 2023; Y. Zhang et al., 2023). It is likely that the high SNR of modern sensors will translate to high pSNR in two-photon neural recordings. This superior signal fidelity should more effectively allow our pipeline to accurately process modern neural recordings with small numbers of ground truth labels.

Finally, we tested how different imaging conditions (e.g., pSNR variability) affected the generalizability of SAND (Extended Data Fig. 4-5). We found that SAND generalized well when the training and test data had similar imaging conditions. For example, SAND trained on the ABO 175 µm dataset and tested on the ABO 275 µm dataset performed as well as SAND trained on the ABO 275 µm dataset and tested on the ABO 275 µm dataset. We then tested ABO-trained SAND on the K53 dataset, which has higher average pSNR values and higher pSNR variability than the ABO dataset. We found that ABO-trained SAND still outperformed CaImAn and Suite2p on K53, but K53-trained SAND achieved the highest accuracy across all numbers of training labels. The accuracy of SAND and SUNS trained on the ABO 275 µm dataset and tested on the K53 dataset decreased as the number of ABO labels used to train these models increased. This is likely the result of increased model specificity when trained on data specific to certain imaging conditions. Augmenting the training data of SAND to make consistent predictions on a variety of noise levels would likely improve model generalizability. For example, we could add an additional training step to SAND to include mutual consistency learning: we could train SAND to predict the same probability maps after adding different amounts of noise to the same frame.

## Discussion

Current methods of neuron segmentation have a trade-off between accuracy and manual effort: supervised methods have superior accuracy but require substantial manual effort to generate ground truth labels for each imaging condition (Abbas and Masip, 2022). This work developed SAND, the first semi-supervised pipeline to segment active neurons from two-photon calcium recordings with limited ground truth labels. SAND effectively operated in this low label regime by using neural network ensembling and a new hyperparameter optimization pipeline. The former process generated a large and robust set of pseudolabels that trained a deep learning segmentation algorithm, while the latter process determined postprocessing hyperparameters from limited numbers of ground truth labels.

SAND achieved higher accuracy than the accuracy of fully supervised methods at multiple scales of labeling. At the small scale, SAND trained on labels from <1% of frames and 25% of all ground truth labels available in a movie was comparably accurate as fully supervised methods trained on all labels. When trained on all available ground truth labels in our movies (>200 neurons), SAND attained higher accuracy than that of current methods. SAND trained on low number of ground truth labels also consistently outperformed matrix factorization methods.

The high accuracy of SAND trained on low numbers of manual labels could allow researchers to circumvent the accuracy–effort trade-off. SAND attained state-of-the-art accuracy with ∼25% of the manual labels, but likely even lower fractions of labeling effort. Previous studies on supervised methods required the manual labeling of all hundreds to thousands of neurons in a single video to serve as a comprehensive training set (Soltanian-Zadeh et al., 2019; Bao et al., 2021). We estimate that manual labelers could identify and outline a single neuron per minute, with diminishing speed as they find fewer neurons when scanning through more frames of a movie. Therefore, SAND could greatly reduce the labeling time needed to generate effective labels for training deep learning neuron segmentation algorithms to well under 1 h per experimental condition.

Pseudolabel training and FLHO both played a role in SAND’s high accuracy when trained on few labels. Pseudolabeling generated a robust training dataset much larger than the manually labeled training set. This larger training set helped train our shallow U-net to distinguish between noise and active neurons, reducing the number of false-positive calls. On the other hand, FLHO improved accuracy by improving hyperparameters in postprocessing. Selection of hyperparameters can greatly impact algorithm performance, but many other pipelines, such as SUNS, CaImAn, and Suite2p, employ supervised postprocessing steps that require large numbers of ground truth labels to accurately tune hyperparameters (Pachitariu et al., 2017; Giovannucci et al., 2019; Bao et al., 2021). FLHO helped bypass the accuracy–effort trade-off in hyperparameter optimization through direct calculation of certain parameters using the limited ground truth labels.

The relationship between the number of ground truth labels and accuracy of neuron segmentation displayed three trends. First, in the regime of extremely low numbers of labels, such as 20–50, SAND outperformed its fully supervised sibling SUNS. Second, both algorithms increased *F*_1_ performance as the number of training labels increased, often reaching performance asymptotes at high numbers of labels ranging from 150 to 250 labels. This large number of labels needed to saturate SAND and SUNS highlights the need for large sets of publicly available manual annotations for a variety of data, such that the field can better understand the conditions that saturate neural network-based segmentations. Third, precision often lagged recall in both SUNS and SAND; the increase in precision largely accounted for the increase in *F*_1_. The reason for this is likely twofold. First, our ensemble learning method averaged the predictions of three models to generate pseudolabels that were conservative and thus reduced training on samples near the detection threshold that could increase false positives. Second, FLHO was also likely conservative. It produced hyperparameters, such as *p_thresh* values that were higher than those found by grid search on the few-label dataset, which eliminated weakly confident predictions.

SAND reduces the manual labeling effort compared with fully supervised algorithms but inherits the prediction speed of the underlying SUNS shallow U-Net architecture (Extended Data Fig. 2-4). This speed was an order of magnitude faster than the rate of data collection (Bao et al., 2021). Fast prediction speed can enable researchers to quickly identify neurons of interest from their recordings in real time and perform targeted perturbation experiments within the same imaging session or during imaging. This capability could help researchers study neural ensemble dynamics in memory and perception that are consistent on the minutes timescale but change from one day to the next (Ziv et al., 2013; Driscoll et al., 2017; Rule et al., 2020; Deitch et al., 2021; Pérez-Ortega et al., 2021). Our ensemble training and hyperparameter optimization processes also reduce training time compared with SUNS because it trained on only 10–25 labeled frames, far fewer than the 1,800 frames used for SUNS. The above benefits at training and test time could also arise from partnerships between existing or future neuron segmentation algorithms and our semi-supervised approaches. Because our ensemble learning and FLHO modify the training approach without dictating the underlying supervised machine learning architecture, these training approaches could retain the accuracy or speed of other algorithms while boosting the other algorithms’ performance in the low label regime.

Similar to all machine learning neuron segmentation algorithms, SAND will likely benefit from recent developments in protein engineering and video processing. Our work showed that SAND in particular benefits from higher response and small variance in response. Such distributional changes have been instantiated by recent generations of protein calcium indicators, which are both more responsive and more linear (Dana et al., 2019; Y. Zhang et al., 2023). Additionally, the development of novel unsupervised video denoising pipelines, such as DeepInterp (Lecoq et al., 2021) and DeepCAD-RT (Li et al., 2022), may also improve recall by reducing noise, thereby increasing SNR. Increases in pSNR have correlated with increased recall (Soltanian-Zadeh et al., 2019; Bao et al., 2021). SNR gains will likely increase precision as well by reporting even small calcium fluctuations.

Future work could directly improve our implementation of SAND or create alternative implementations. Direct improvement of SAND could optimize the frame selection or model selection to maximize accuracy. Our current approach randomly selected the frames used for labeling. It is possible that systematic selection of these frames could more effectively represent the range of neuron characteristics (e.g., size and pSNR) with even fewer ground truth labels. Additionally, our current approach defaulted to the SUNS shallow U-net architecture as the final neural network to make neuron predictions. Future iterations of SAND could evaluate the accuracy of all ensemble U-nets when processing the ground truth data and then perform pseudolabel training on the U-net with the lowest error. Finally, improvements to SAND or SUNS could also help detect neurons by improving the postprocessing classification step. Such changes could use dynamic information from a large temporal extent to detect sparsely and weakly active neurons (Soltanian-Zadeh et al., 2019).

Application of SAND beyond the two-photon datasets in this work are potentially numerous. Future SAND applications could help process imaging data from one-photon or volumetric imaging settings, which generally have lower SNR than planar two-photon imaging (Jung et al., 2004; Ahrens et al., 2013; Ji et al., 2016; Waters, 2020). SAND can stand alone to process such data or pair with segmentation algorithms that target specific optical imaging data types (Yuanlong Zhang et al., 2023). Likewise, future testing could also apply SAND to process the diverse calcium recordings of many cell types, such as inhibitory neurons or glia (Akerboom et al., 2013; Semyanov et al., 2020; Mulholland et al., 2021). SAND’s ability to accurately segment neurons in the few labels regime can potentially help individual labs process imaging data from distinctive imaging preparations even if a substantial manually labeled training dataset, generated by a single lab or large community, does not yet exist.

## Synthesis

Reviewing Editor: Sam Golden, The University of Washington Decisions are customarily a result of the Reviewing Editor and the peer reviewers coming together and discussing their recommendations until a consensus is reached. When revisions are invited, a fact-based synthesis statement explaining their decision and outlining what is needed to prepare a revision will be listed below. The following reviewer(s) agreed to reveal their identity: NONE.

Dear Authors,

Two reviewers have reviewed the manuscript and provided a congruent set of recommendations, including that the revisions are straightforward enough to need only editorial oversight upon resubmission.

Overall, the reviewers find the SAND package to potentially useful to the field. Both reviewers and myself would like the complete code/package to be available on Github (or similar host) prior to final manuscript approval.

Below please find the reviewer comments that require a point-by-point rebuttal:

Reviewer 1:

Some comments on clarification.

(1) What is the decision process of using current three variants of U-Net architectures with different decoders, but not other variants.

(2) Plotting learning curves across epochs for training and test dataset will be informative for reader about the fitting process.

(3) Plot an example grid search distribution of different centroid_dist and min_area values that maximize the F1 score. Does different p_thresh values at intermediate steps result in the same centroid_dist and min_area values, which explains the accuracy invariance to the p_thresh?

(4) Direct comparison of training and test time for SAND, in comparison to SUNS which requires more training data but simpler network.

Reviewer 2:

Major

• Can the authors explain the rationale for using three neural networks for pseudolabel generation and the rationale for how each individual NN was structured. How is this specific architecture important for processing the biological data?

• Figures 2-4 show robust results of SUNS outperforming other algorithms with lower number of labelled neurons. It would also be helpful to plot performance as a function of number of frames (with average number of neurons per frame locked to around 100-150 cells) - this can provide a sense to readers how many frames they expect to prepare.

• As data collected on 2-photon microscopes can vary in noise levels, it would be useful to assess the algorithm's performance on data with more extreme noise regimes (the SNR in the analyzed datasets are generally high SNR). For example, the authors could generate or modify data to cover a range of 4 to 5 SNR levels in a similar fashion as Rupprecht et al. (Nat Neurosci. 2021).

• How well does the SAND algorithm generalize from one distinct dataset to another. Given that algorithms like suite2p can accommodate different datasets (cortical and subcortical, 1x versus 4x zoom, etc.) during ROI detection without explicit training, it would be helpful to know if such generalizability is a feature of SAND. In other words, if SAND was trained on the ABO V1 dataset, does it perform similarly well on the CaImAn V1 dataset.

• Can the authors speak about the time to train the models and generate pseudolabels, and time to complete postprocessing? How does this total time compare to the time to complete analyses from competing algorithms?

Minor

• Significance statement: is SAND an acronym? I suggest that it is spelled out when it's first mentioned.

• Methods - preprocessing: please specify details on how temporal filtering was performed (i.e. cutoff frequency, filter type)

• Methods - model training: please provide conceptual rationale for why you chose 3 different U-nets for pseudolabel generation

• Methods - model training: The authors refer to the labeled data going into the initial 3 U-net training step as "epochs". What is meant by this? How many individually labeled frames went into this step of training?

• Methods - hyperparameter optimization: It would be informative to readers to see what the 25% and median p_thresh probability maps look like. Such a panel might provide insight to which ROIs are being considered in each FLHO step

• When making statements about accuracy of algorithm generated ROIs shape relative to ground truth, it would be useful to include metrics (like jaccard index) and statistics.

• When making statements about significance, please include either the statistics in the text or refer to the table to find relevant statistics. One example is this sentence: "Additionally, the F1 score for SAND when trained on more than 250 neurons was significantly higher than the SUNS F1 score"

• Please provide data on the performance of pseudolabel generation and comparing the probability maps to the groundtruth.

• In the text, the SAND pipeline involves FLHO optimization; however, in figures such as 2, it's not clear if FLHO is included in the SAND measurements. Please consider replacing "SAND" label to SAND+FLHO"

## References

[B1] Abbas W, Masip D (2022) Computational methods for neuron segmentation in two-photon calcium imaging data: a survey. Appl Sci 12:6876. 10.3390/app12146876

[B2] Ahrens MB, Orger MB, Robson DN, Li JM, Keller PJ (2013) Whole-brain functional imaging at cellular resolution using light-sheet microscopy. Nat Methods 10:413–420. 10.1038/nmeth.2434 23524393

[B3] Akerboom J, et al. (2012) Optimization of a GCaMP calcium indicator for neural activity imaging. J Neurosci 32:13819–13840. 10.1523/JNEUROSCI.2601-12.2012 23035093 PMC3482105

[B4] Akerboom J, et al. (2013) Genetically encoded calcium indicators for multi-color neural activity imaging and combination with optogenetics. Front Mol Neurosci 6:43920. 10.3389/FNMOL.2013.00002/BIBTEX PMC3586699 23459413

[B5] Bao Y, Gong Y (2023) Machine learning data processing as a bridge between microscopy and the brain. In: *Intelligent nanotechnology: merging nanoscience and artificial intelligence* (Zheng Y, Wu Z, ed), pp 399–420. Elsevier. 10.1016/B978-0-323-85796-3.00014-7

[B6] Bao Y, Redington E, Agarwal A, Gong Y (2022) Decontaminate traces from fluorescence calcium imaging videos using targeted non-negative matrix factorization. Front Neurosci 15:797421. 10.3389/FNINS.2021.797421 35126042 PMC8815790

[B7] Bao Y, Soltanian-Zadeh S, Farsiu S, Gong Y (2021) Segmentation of neurons from fluorescence calcium recordings beyond real-time. Nat Mach Intell 3:590–600. 10.1038/s42256-021-00342-x 34485824 PMC8415119

[B8] Chaitanya K, Erdil E, Karani N, Konukoglu E (2020) Contrastive learning of global and local features for medical image segmentation with limited annotations. Advances in Neural Information Processing Systems, 2020-Decem, 12546–12558.

[B9] Chen TW, et al. (2013) Ultra-sensitive fluorescent proteins for imaging neuronal activity. Nature 499:295. 10.1038/NATURE12354 23868258 PMC3777791

[B30] CodeNeuro (2016) *The neurofinder challenge*.

[B10] Dana H, et al. (2019) High-performance calcium sensors for imaging activity in neuronal populations and microcompartments. Nat Methods 16:649–657. 10.1038/s41592-019-0435-6 31209382

[B11] Deitch D, Rubin A, Ziv Y (2021) Representational drift in the mouse visual cortex. Curr Biol 31:4327–4339.e6. 10.1016/j.cub.2021.07.062 34433077

[B12] Demas J, Manley J, Tejera F, Kim H, Traub FM, Chen B, Vaziri A (2021) High-speed, cortex-wide volumetric recording of neuroactivity at cellular resolution using light beads microscopy. *BioRxiv*, 2021.02.21.432164. 10.1101/2021.02.21.432164 PMC8958902 34462592

[B13] de Vries SEJ, et al. (2020) A large-scale standardized physiological survey reveals functional organization of the mouse visual cortex. Nat Neurosci 23:138–151. 10.1038/S41593-019-0550-9 31844315 PMC6948932

[B14] Dietterich T (2002) *The handbook of brain theory and neural networks*. Cambridge, MA: MIT Press.

[B15] Driscoll LN, Pettit NL, Minderer M, Chettih SN, Harvey CD (2017) Dynamic reorganization of neuronal activity patterns in parietal cortex. Cell 170:986–999.e16. 10.1016/J.CELL.2017.07.021 28823559 PMC5718200

[B16] Giovannucci A, et al. (2019) Caiman an open source tool for scalable calcium imaging data analysis. eLife 8:e38173. 10.7554/eLife.38173 30652683 PMC6342523

[B17] Grienberger C, Konnerth A (2012) Imaging calcium in neurons. Neuron 73:862–885. 10.1016/J.NEURON.2012.02.011 22405199

[B18] Huang W, Chen C, Xiong Z, Zhang Y, Chen X, Sun X, Wu F (2022) Semi-supervised neuron segmentation via reinforced consistency learning. IEEE Trans Med Imaging 41:3016–3028. 10.1109/TMI.2022.3176050 35584076

[B19] Ji N, Freeman J, Smith SL (2016) Technologies for imaging neural activity in large volumes. Nat Neurosci 19:1154–1164. 10.1038/nn.4358 27571194 PMC5244827

[B20] Jung JC, Mehta AD, Aksay E, Stepnoski R, Schnitzer MJ (2004) In vivo mammalian brain imaging using one- and two-photon fluorescence microendoscopy. J Neurophysiol 92:3121. 10.1152/JN.00234.2004 15128753 PMC2826362

[B21] Keemink SW, Lowe SC, Pakan JMP, Dylda E, Van Rossum MCW, Rochefort NL (2018) FISSA: a neuropil decontamination toolbox for calcium imaging signals. Sci Rep 8:1–12. 10.1038/s41598-018-21640-2 29472547 PMC5823956

[B22] Krizhevsky A, Sutskever I, Hinton GE (2017) ImageNet classification with deep convolutional neural networks. Commun ACM 60:84–90. 10.1145/3065386

[B23] Lecoq J, Oliver M, Siegle JH, Orlova N, Ledochowitsch P, Koch C (2021) Removing independent noise in systems neuroscience data using DeepInterpolation. Nat Methods 18:1401–1408. 10.1038/s41592-021-01285-2 34650233 PMC8833814

[B24] Lee D-H (2013) Pseudo-label: The simple and efficient semi-supervised learning method for deep neural networks. *Workshop on Challenges in Representation Learning, ICML*, *3*(2), 896.

[B25] Li X, et al. (2022) Real-time denoising enables high-sensitivity fluorescence time-lapse imaging beyond the shot-noise limit. Nat Biotechnol 41:282–292. 10.1038/s41587-022-01450-8 36163547 PMC9931589

[B26] Makino H, Ren C, Liu H, Kim AN, Kondapaneni N, Liu X, Kuzum D, Komiyama T (2017) Transformation of cortex-wide emergent properties during motor learning. Neuron 94:880–890.e8. 10.1016/J.NEURON.2017.04.015 28521138 PMC5502752

[B27] Mulholland HN, Hein B, Kaschube M, Smith GB (2021) Tightly coupled inhibitory and excitatory functional networks in the developing primary visual cortex. eLife 10:e72456. 34878404 10.7554/eLife.72456 PMC8654369

[B28] Muller D, Soto-Rey I, Kramer F (2022) An analysis on ensemble learning optimized medical image classification with deep convolutional neural networks. IEEE Access 10:66467–66480. 10.1109/ACCESS.2022.3182399

[B29] Nakai J, Ohkura M, Imoto K (2001) A high signal-to-noise Ca(2+) probe composed of a single green fluorescent protein. Nat Biotechnol 19:137–141. 10.1038/84397 11175727

[B31] Ouali Y, Hudelot C, Tami M (2020) An overview of deep semi-supervised learning. *ArXiv*.

[B32] Pachitariu M, Stringer C, Dipoppa M, Schröder S, Rossi LF, Dalgleish H, Carandini M, Harris KD (2017) Suite2p: beyond 10,000 neurons with standard two-photon microscopy. *BioRxiv*. 10.1101/061507

[B33] Pérez-Ortega J, Alejandre-García T, Yuste R (2021) Long-term stability of cortical ensembles. eLife 10:e64449. 10.7554/eLife.64449 34328414 PMC8376248

[B34] Pnevmatikakis EA, Giovannucci A (2017) NoRMCorre: an online algorithm for piecewise rigid motion correction of calcium imaging data. J Neurosci Methods 291:83–94. 10.1016/j.jneumeth.2017.07.031 28782629

[B35] Pnevmatikakis EA, Soudry D, Gao Y, Peterka DS, Yuste R, Correspondence LP (2016) Simultaneous denoising, deconvolution, and demixing of calcium imaging data. Neuron 89:285–299. 10.1016/j.neuron.2015.11.037 26774160 PMC4881387

[B36] Polikar R (2006) Ensemble based systems in decision making. IEEE Circuits Syst Mag 6:21–44. 10.1109/MCAS.2006.1688199

[B37] Rule ME, Loback AR, Raman DV, Driscoll LN, Harvey CD, O’leary T (2020) Stable task information from an unstable neural population. eLife 9:e51121. 10.7554/eLife.38173 32660692 PMC7392606

[B38] Rumyantsev OI, Lecoq JA, Hernandez O, Zhang Y, Savall J, Chrapkiewicz R, Li J, Zeng H, Ganguli S, Schnitzer MJ (2020) Fundamental bounds on the fidelity of sensory cortical coding. Nature 580:100–105. 10.1038/S41586-020-2130-2 32238928

[B39] Ryan MB, Churchland AK, Gong Y, Baker C (2023) Fastest-ever calcium sensors broaden the potential of neuronal imaging. Nature 615:804–805. 10.1038/d41586-023-00704-y 36922656

[B40] Sagi O, Rokach L (2018) Ensemble learning: a survey. Wiley Interdiscip Rev: Data Min Knowl Discov 8:e1249. 10.1002/widm.1249

[B41] Semyanov A, Henneberger C, Agarwal A (2020) Making sense of astrocytic calcium signals — from acquisition to interpretation. Nat Rev Neurosci 21:551–564. 10.1038/s41583-020-0361-8 32873937

[B42] Sohn K, Berthelot D, Li CL, Zhang Z, Carlini N, Cubuk ED, Kurakin A, Zhang H, Raffel C (2020) FixMatch: Simplifying semi-supervised learning with consistency and confidence. Advances in Neural Information Processing Systems, 2020-Decem.

[B43] Soltanian-Zadeh S, Sahingur K, Blau S, Gong Y, Farsiu S (2019) Fast and robust active neuron segmentation in two-photon calcium imaging using spatiotemporal deep learning. Proc Natl Acad Sci U S A 116:8554–8563. 10.1073/pnas.1812995116 30975747 PMC6486774

[B44] Stevenson IH, Kording KP (2011) How advances in neural recording affect data analysis. Nat Neurosci 14:139–142. 10.1038/nn.2731 21270781 PMC3410539

[B45] Stosiek C, Garaschuk O, Holthoff K, Konnerth A (2003) In vivo two-photon calcium imaging of neuronal networks. Proc Natl Acad Sci U S A 100:7319–7324. 10.1073/PNAS.1232232100/ASSET/B3D9FDB8-4437-42E4-8058-8AADAF92B00B/ASSETS/GRAPHIC/PQ1232232005.JPEG 12777621 PMC165873

[B46] Stringer C, Pachitariu M, Steinmetz N, Carandini M, Harris KD (2019) High-dimensional geometry of population responses in visual cortex. Nature 571:361–365. 10.1038/S41586-019-1346-5 31243367 PMC6642054

[B47] Sweis BM, Mau W, Rabinowitz S, Cai DJ (2021) Dynamic and heterogeneous neural ensembles contribute to a memory engram. Curr Opin Neurobiol 67:199–206. 10.1016/J.CONB.2020.11.017 33388602 PMC8192335

[B48] Theis L, Berens P, Froudarakis E, Reimer J, Román Rosón M, Baden T, Euler T, Tolias AS, Bethge M (2016) Benchmarking spike rate inference in population calcium imaging. Neuron 90:471–482. 10.1016/J.NEURON.2016.04.014/ATTACHMENT/262BE9B0-ACBE-4015-8BC3-C21A1BA8AF1D/MMC1.PDF 27151639 PMC4888799

[B49] Tian L, et al. (2009) Imaging neural activity in worms, flies and mice with improved GCaMP calcium indicators. Nat Methods 6:875–881. 10.1038/nmeth.1398 19898485 PMC2858873

[B50] Vyas S, Golub MD, Sussillo D, Shenoy KV (2020) *Computation through neural population dynamics*. 10.1146/annurev-neuro-092619 PMC7402639 32640928

[B51] Waters J (2020) Sources of widefield fluorescence from the brain. ELife 9:1–13. 10.7554/ELIFE.59841 PMC7647397 33155981

[B52] Wu Y, Ge Z, Zhang D, Xu M, Zhang L, Xia Y, Cai J (2022) Mutual consistency learning for semi-supervised medical image segmentation. Med Image Anal 81:102530. 10.1016/J.MEDIA.2022.102530 35839737

[B53] Yuste R (2015) From the neuron doctrine to neural networks. Nat Rev Neurosci 16:487–497. 10.1038/nrn3962 26152865

[B54] Zhang Y, et al. (2023) Fast and sensitive GCaMP calcium indicators for imaging neural populations. Nature 615:884–891. 10.1038/s41586-023-05828-9 36922596 PMC10060165

[B55] Zhang L, Tanno R, Xu MC, Jin C, Jacob J, Ciccarelli O, Barkhof F, Alexander DC (2020) Disentangling human error from the ground truth in segmentation of medical images. Advances in Neural Information Processing Systems, 2020-Decem.

[B56] Zhang Yuanlong, Zhang G, Han X, Wu J, Li Z, Li X, Xiao G, Xie H, Fang L, Dai Q (2023) Rapid detection of neurons in widefield calcium imaging datasets after training with synthetic data. Nat Methods 20:747–754. 10.1038/s41592-023-01838-7 37002377 PMC10172132

[B57] Zheng H, Zhang Y, Yang L, Liang P, Zhao Z, Wang C, Chen DZ (2019) A new ensemble learning framework for 3D biomedical image segmentation. Proceedings of the AAAI Conference on Artificial Intelligence, 33(01), 5909–5916. 10.1609/AAAI.V33I01.33015909

[B58] Zhuang C, Yan S, Nayebi A, Schrimpf M, Frank MC, DiCarlo JJ, Yamins DLK (2021) Unsupervised neural network models of the ventral visual stream. Proc Natl Acad Sci U S A 118:e2014196118. 10.1073/PNAS.2014196118 33431673 PMC7826371

[B59] Ziv Y, Burns LD, Cocker ED, Hamel EO, Ghosh KK, Kitch LJ, El Gamal A, Schnitzer MJ (2013) Long-term dynamics of CA1 hippocampal place codes. Nat Neurosci 16:264–266. 10.1038/nn.3329 23396101 PMC3784308

[B60] Zou Y, Zhang Z, Zhang H, Li C-L, Bian X, Huang J-B, Pfister T (2020) PseudoSeg: designing pseudo labels for semantic segmentation. *ArXiv*.

