# Supplementary figures and images for: A Semi-supervised Pipeline for Accurate Neuron Segmentation with Fewer Ground Truth Labels
Source: eNeuro. 2024 Feb 9;11(2):ENEURO.0352-23.2024. doi: 10.1523/ENEURO.0352-23.2024 (PMC10880440; doi:10.1523/ENEURO.0352-23.2024)

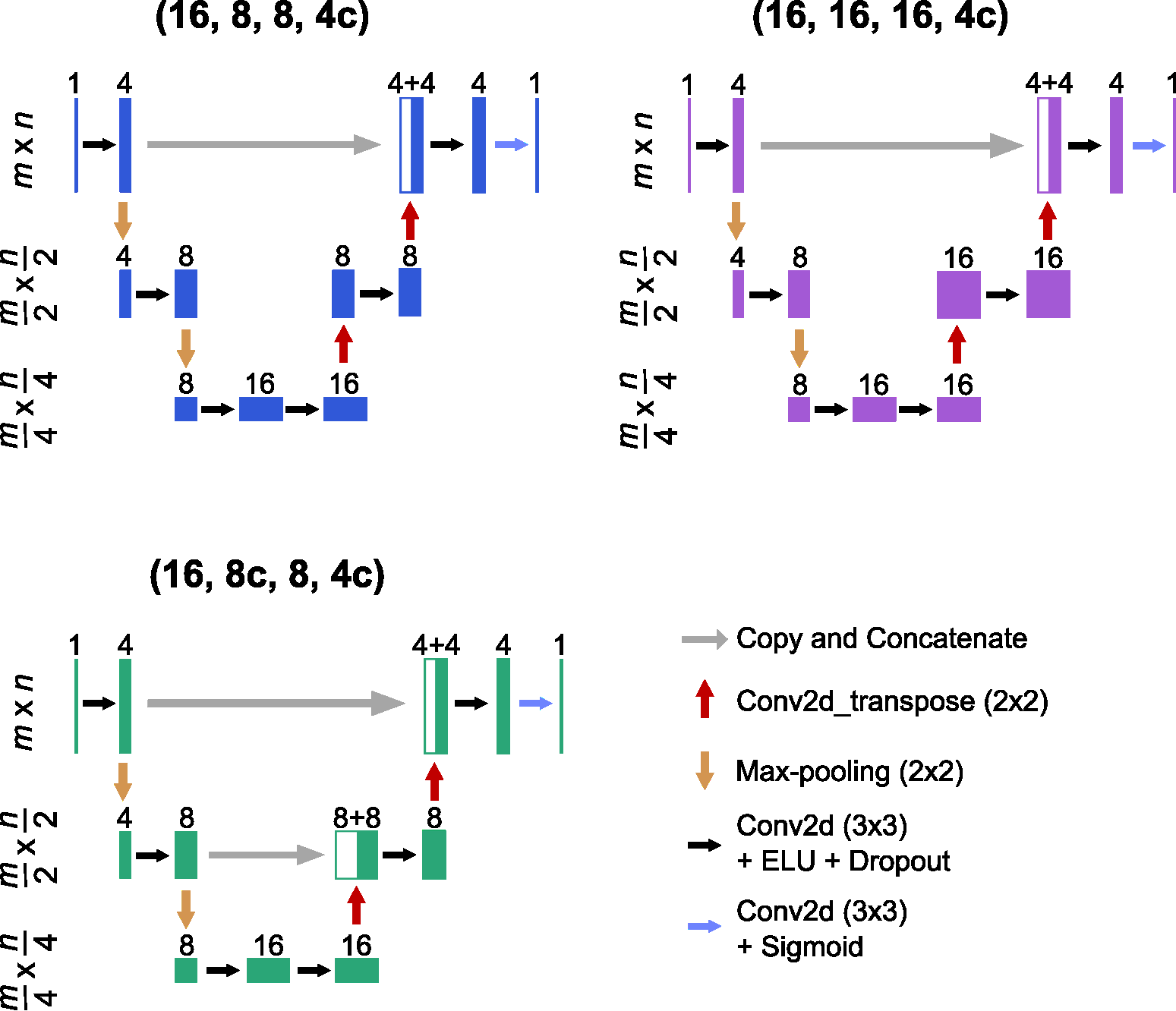

Supplement: Figure 1-1 — Multiple neural network architectures generated the pseudolabels and final predictions. We used three U-Net architectures with the same encoder but varying decoders. The labels above the U-Nets represent the number of channels in each level of the decoder, starting with the deepest layer, and ‘c' denotes a concatenation. The numbers above each block represent the number of channels at each layer and the variables to the left represent the dimensions of the image at each step, given an input frame with dimensions m × n. We used a dropout rate of 0.1 for the first two depths and a rate of 0.2 for the deepest depth. The total number of trained parameters for each architecture was ∼5000 (blue), ∼6000 (green), and ∼7500 (purple). Download Figure 1-1, TIF file. [file eneuro-11-ENEURO.0352-23.2024-s002.tif]

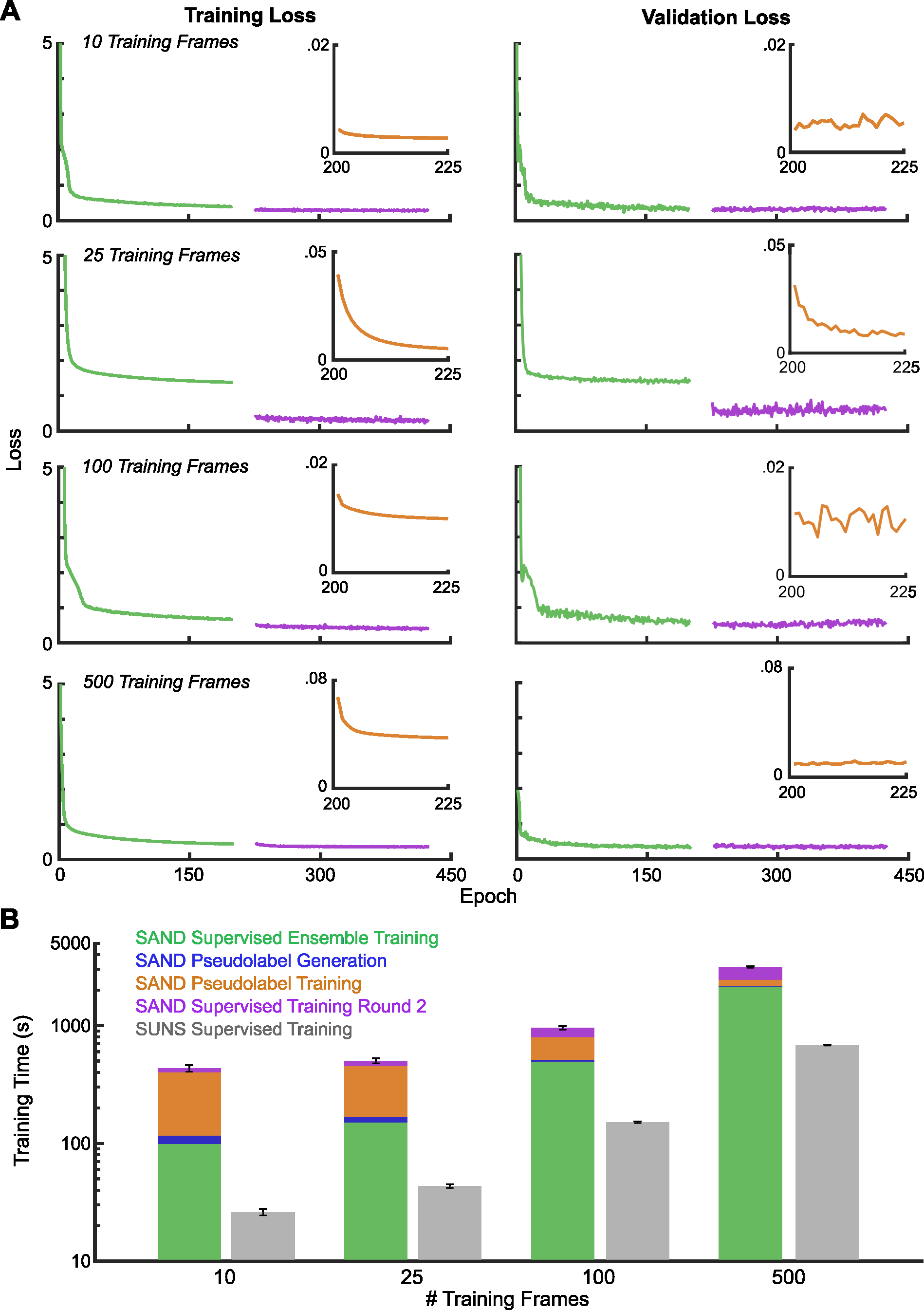

Supplement: Figure 1-2 — Pseudolabel training helped reduce and stabilize training loss. (A) Example learning curves for training and validation sets using different amounts of training labels on ABO 275 µm data. The first round of supervised training (green) lasted for 200 epochs. We then trained models of pseudolabels (orange) for 25 epochs. A final round of training on the labeled frames (purple) lasted for another 200 epochs. (Insets) Corresponding learning curves on pseudolabels, which used binary cross entropy loss. (B) Breakdown of training time for SAND and SUNS for each step of training using different amounts of ground truth labels. Total training time for SAND and SUNS increased as the number of training labels increased. Even when trained on 500 frames, both SAND and SUNS could be trained in less than an hour. Bars represent the average training time for 10 different models. Error bars represent the standard deviation for the total training time (n = 10 models). Download Figure 1-2, TIF file. [file eneuro-11-ENEURO.0352-23.2024-s003.tif]

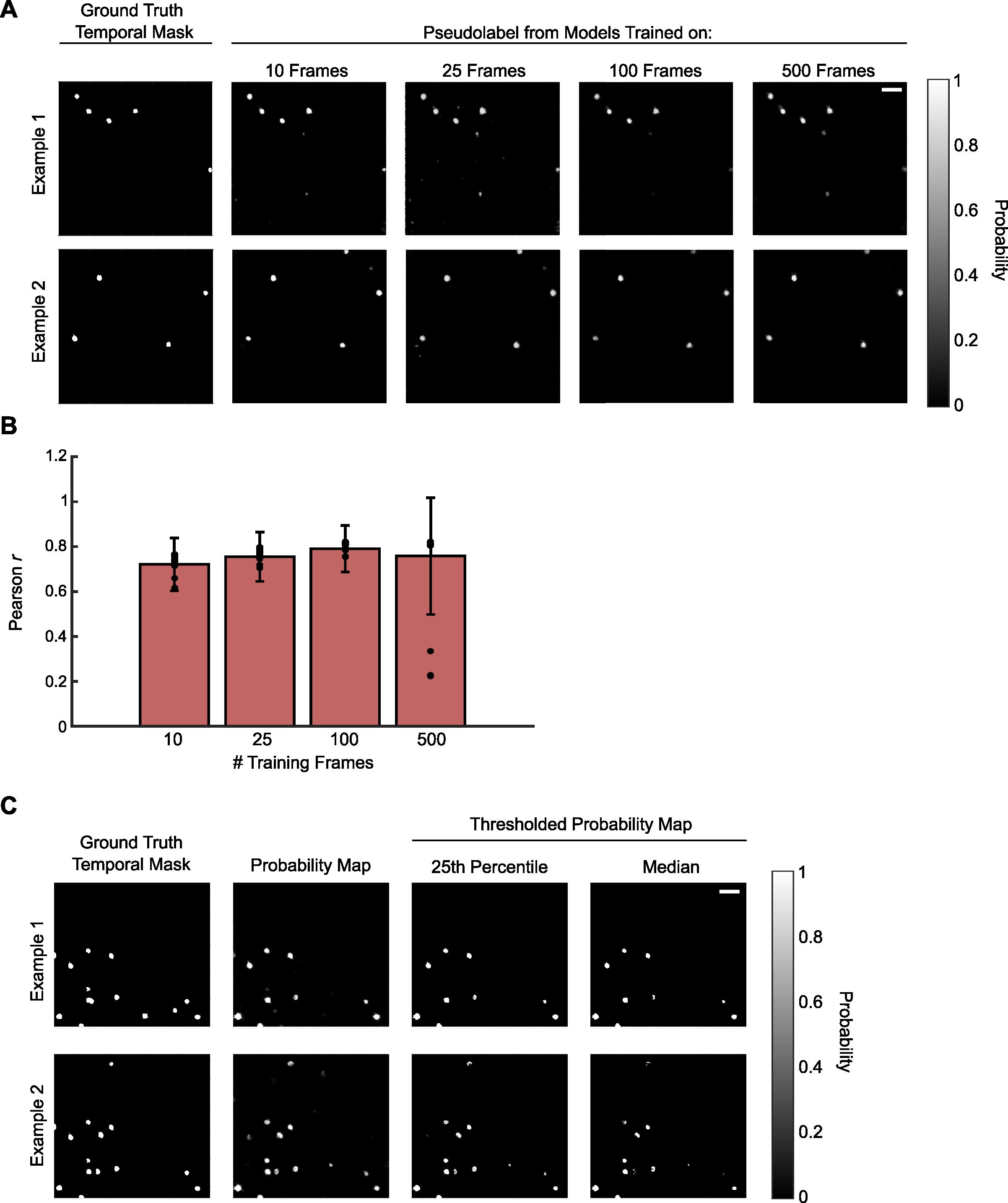

Supplement: Figure 1-3 — Pseudolabels and final probability maps closely aligned with ground truth temporal masks. (A) Example pseudolabels for two different ABO 275 μm frames generated by ensembles trained on different numbers of labeled frames. Pseudolabels were generally consistent across different numbers of training frames. The scale bar is 50 μm. (B) Pseudolabels were strongly correlated with the ground truth temporal masks. Bars represent the median correlation values between 1000 pseudolabels and ground truth temporal masks (100 labels from 10 different models). Error bars represent standard deviation. Data points represent the median correlation values for each model. (C) Example probability maps used for FLHO. Probability maps closely aligned with the ground truth temporal mask. Thresholding with the 25th percentile value helped retain lower confidence neurons. The scale bar is 50 μm. Download Figure 1-3, TIF file. [file eneuro-11-ENEURO.0352-23.2024-s004.tif]

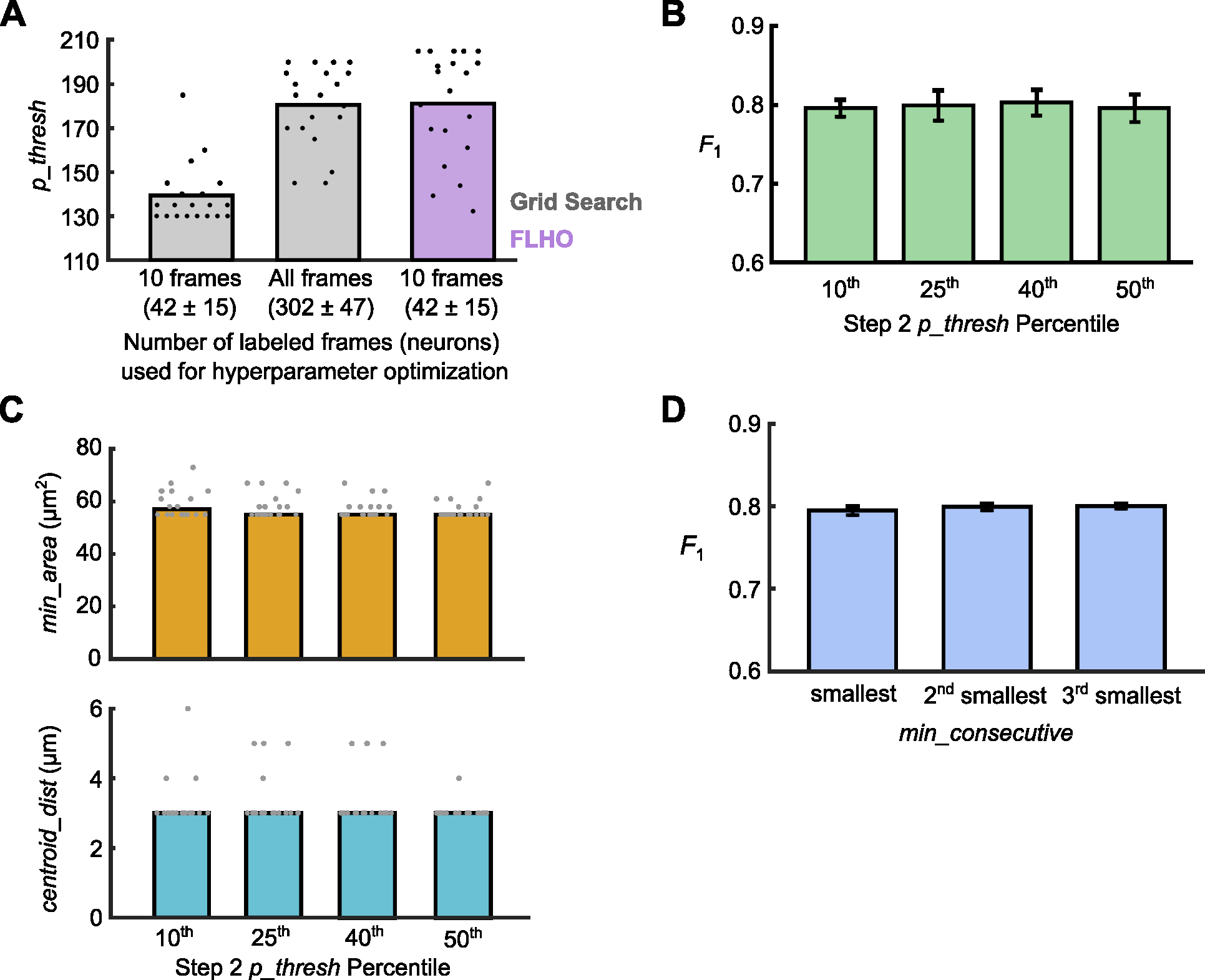

Supplement: Figure 1-4 — Few label hyperparameter optimization accurately estimated SAND hyperparameters and was invariant to optimization choices. (A) Grid searches for hyperparameters with small numbers of ground truth labels consistently underestimated the optimal p_thresh value. We performed a traditional grid search for all hyperparameters on 20 models, each trained on randomly selected sets of 10 frames from the ABO 275 µm dataset (2 models per video). We also used grid search for all hyperparameters using all labels from all frames to generate the ‘optimal' hyperparameters. p_thresh tuned on 10 frames was consistently lower than the optimal p_thresh. We then calculated the hyperparameters for the 20 models using our new hyperparameter optimization method, FLHO. This method produced p_thresh values that were similar to the optimal p_thresh. p_thresh is listed as a grayscale pixel value from 0 to 255 (e.g. a value of 205 corresponds to a probability of 80%). Parentheses show the mean ± standard deviation of the number of labeled neurons used in hyperparameter optimization. (B) The accuracy of our method was robust to small changes in intermediate p_thresh. We performed our hyperparameter optimization pipeline on 20 models using four different percentiles for intermediate p_thresh, used during step 2 of the pipeline. Each model was trained on 10 labeled frames from the ABO 275 µm dataset (73 ± 22 labeled neurons). A one-way Kruskal-Wallis test did not find a significant difference in performance between the four choices of p_thresh (p = 0.20). Bars and error bars respectively represent mean and standard error. (C) The values of centroid_dist and min_area determined by the grid search were robust to changes in the p_thresh percentile used in step 2 of FLHO. Bars represent the median values of centroid_dist and min_area for the models in B. A one-way Kruskal-Wallis test did not find a significant difference in the grid search selection between the four choices of p_thresh for min_area or [file eneuro-11-ENEURO.0352-23.2024-s005.tif]

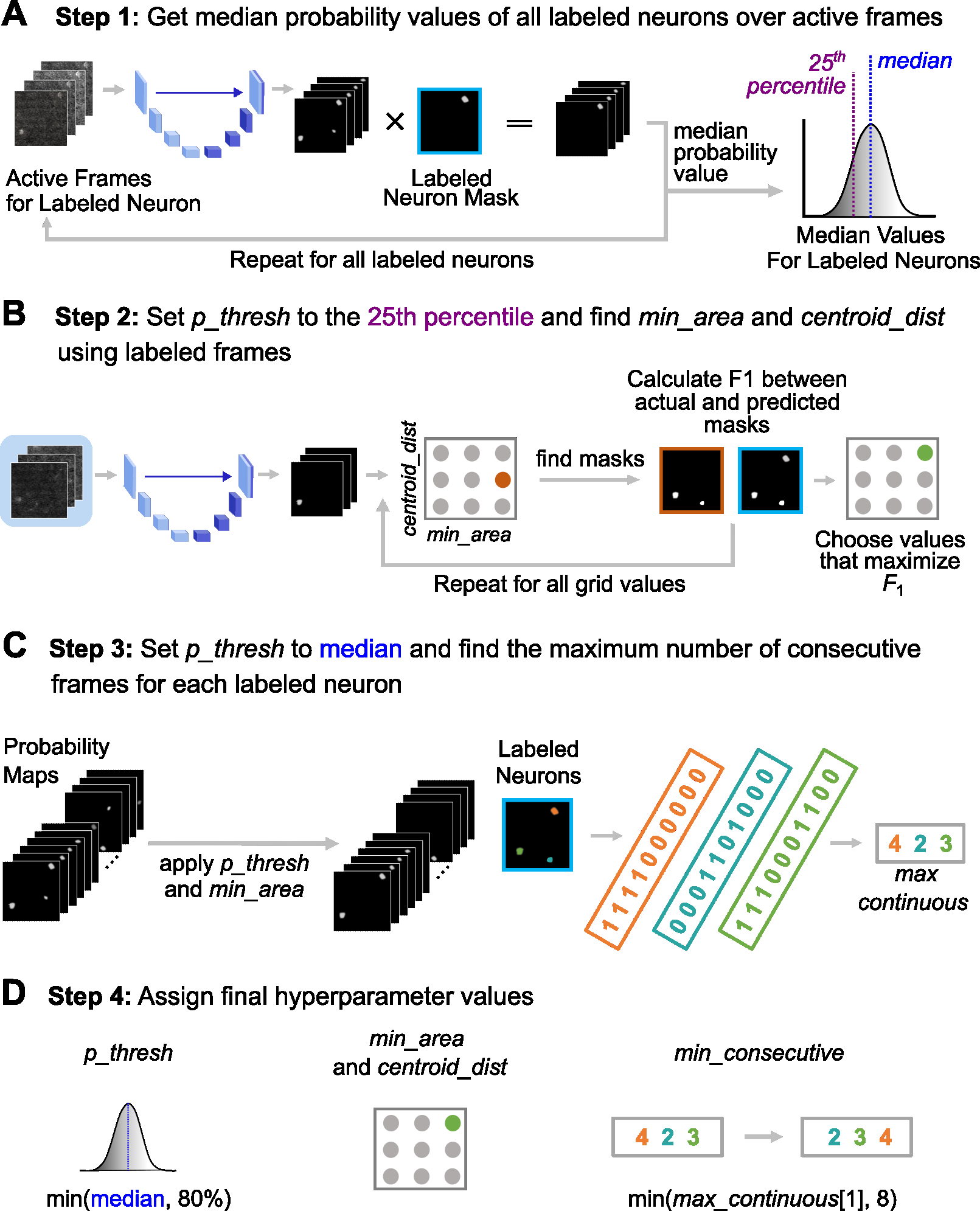

Supplement: Figure 1-5 — The Few Label Hyperparameter Optimization pipeline had four main steps. (A) We determined the 25th percentile and median values for p_thresh. The 25th percentile value was used in Step 2 and the median value was used in steps 3 and 4, which included neuron prediction. (B) We used a grid search to find the min_area and centroid_dist values that maximized F1 on labeled frames. (C) We found the maximum number of continuous frames for each ground truth neuron. (D) We determined the final hyperparameters. We placed an upper bound of 80% on p_thresh. To determine min_consecutive, we found the second smallest number of continuous frames from Step 3. We placed an upper bound of 8 frames on this value. Download Figure 1-5, TIF file. [file eneuro-11-ENEURO.0352-23.2024-s006.tif]

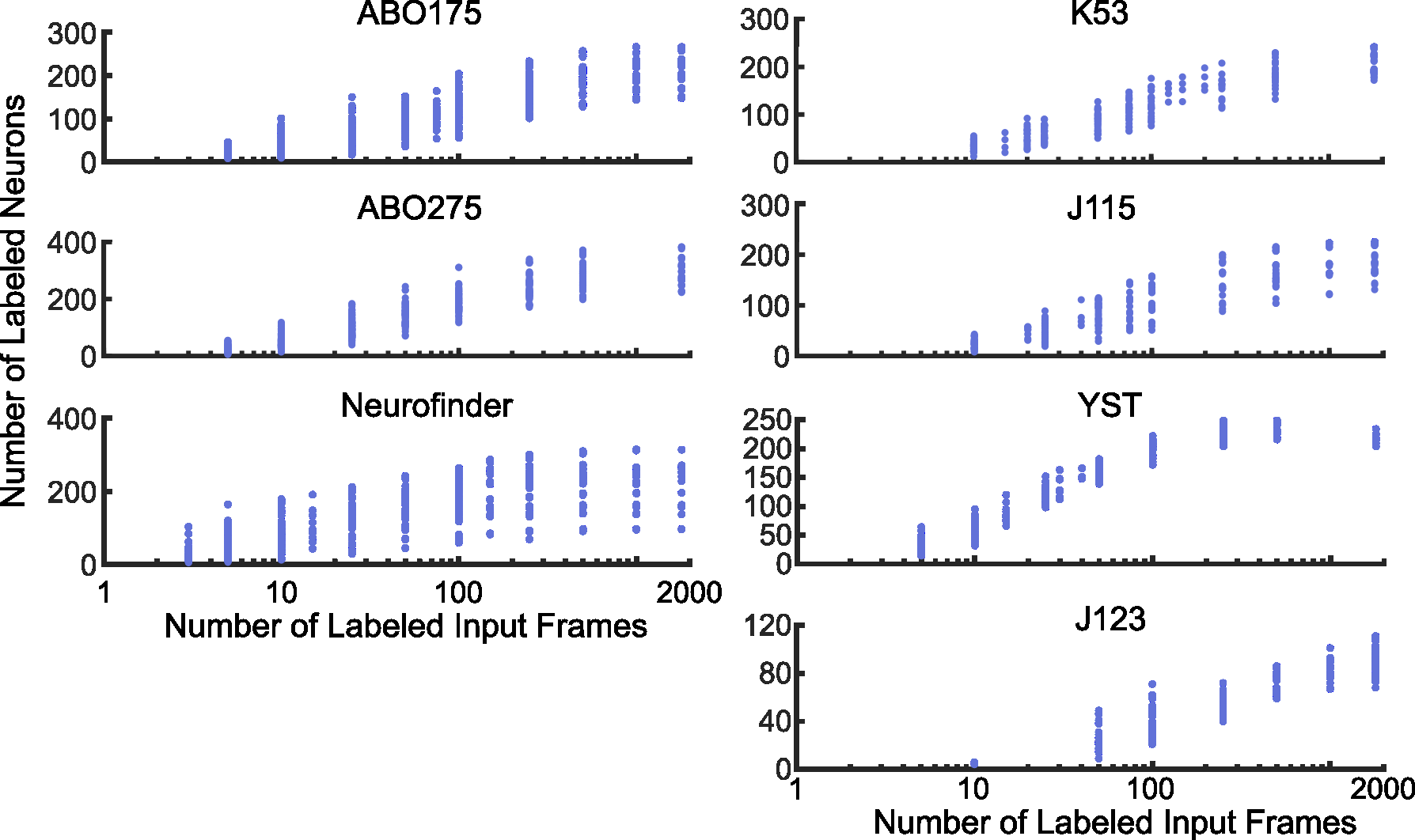

Supplement: Figure 1-6 — We adjusted the number of training labels by randomly sampling different numbers of training frames. A scatter plot of the number of labeled frames vs. number of labeled neurons for each dataset shows that the number of training labels (neurons) increased as we increased the number of training frames. Each point represents a unique model. Download Figure 1-6, TIF file. [file eneuro-11-ENEURO.0352-23.2024-s007.tif]

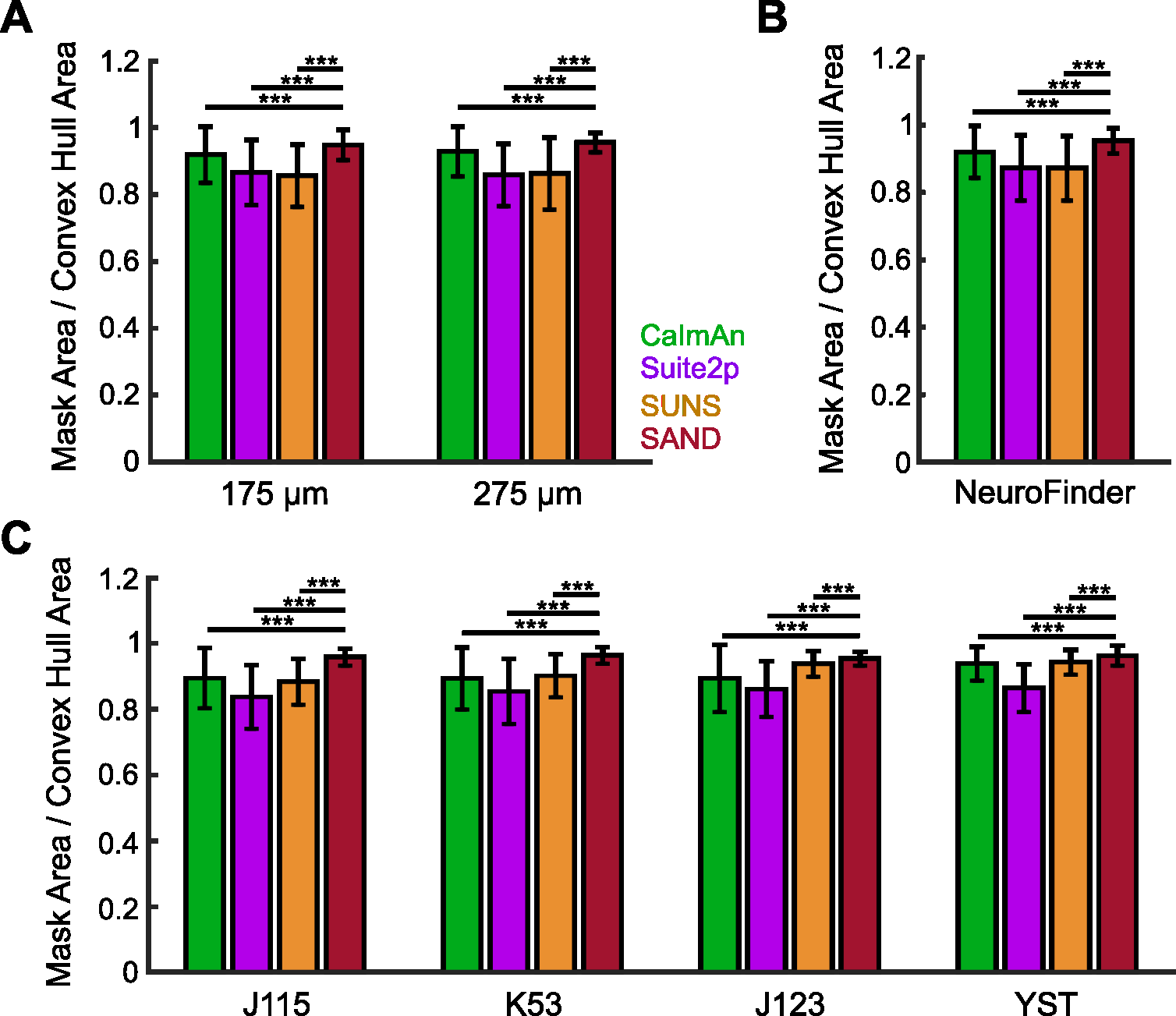

Supplement: Figure 2-1 — SAND had higher quality masks than competing methods. (A) Masks identified by SAND were more consistently shaped like neuron somas than masks identified by other methods on the ABO datasets. SUNS and SAND were both trained on 10 frames. (B) Masks identified by SAND were more consistently shaped like neuron somas than masks identified by other methods on the Neurofinder dataset. SUNS and SAND were both trained on 10 frames. (C) Masks identified by SAND were more consistently shaped like neuron somas than masks identified by other methods on the CaImAn datasets. SUNS and SAND were both trained on 10 frames for J115, K53, and YST. SUNS and SAND were both trained on 100 frames for J123. Bars represent average ratio values and error bars represent standard deviation. *** indicates p < 0.001 (Tables 2-2, 2-3, 2-4). Download Figure 2-1, TIF file. [file eneuro-11-ENEURO.0352-23.2024-s008.tif]

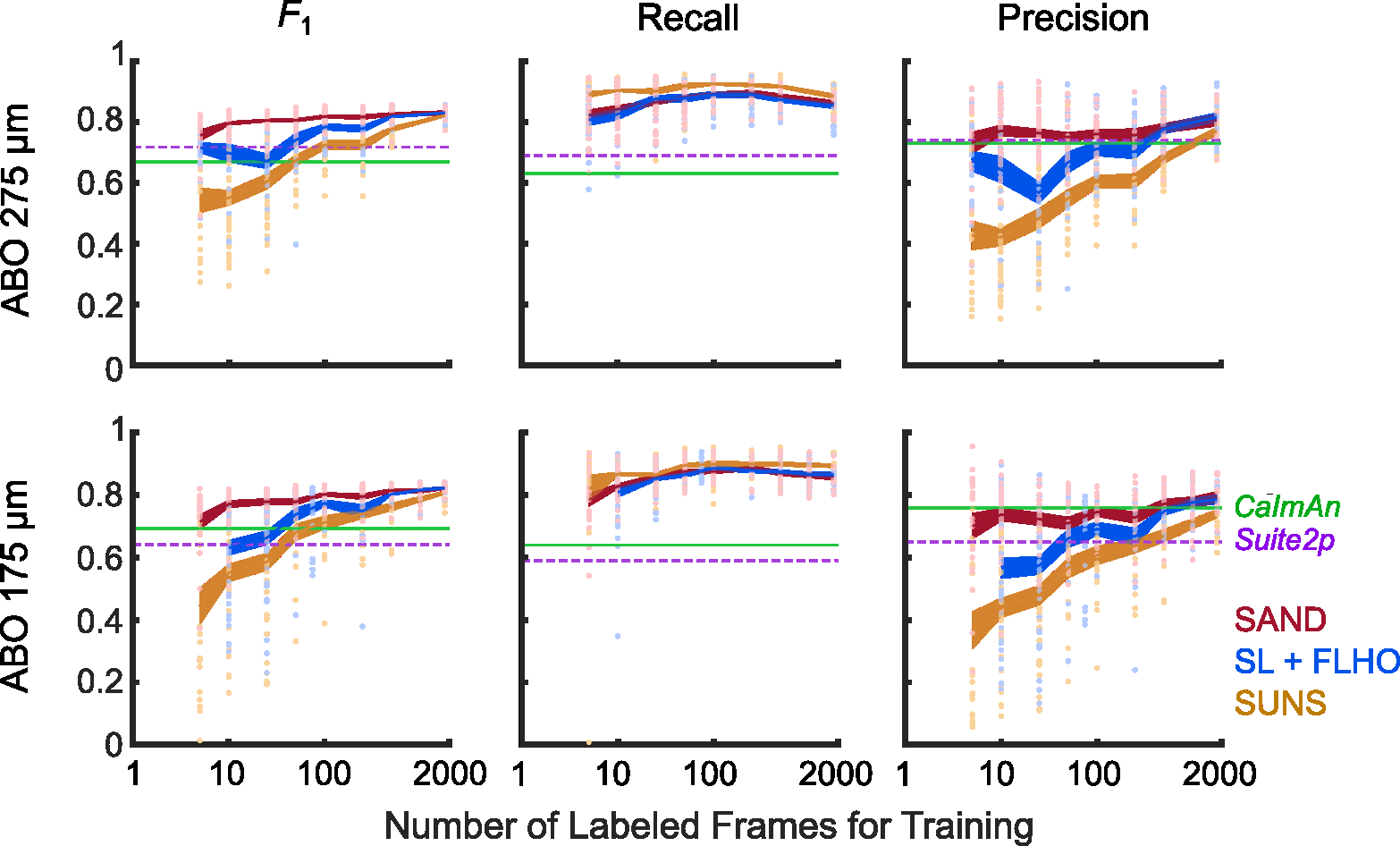

Supplement: Figure 2-2 — SAND outperforms other methods on the ABO dataset when trained on fewer labeled frames. SAND had higher accuracy than other methods with low number of labeled frames on both the ABO 275 μm and ABO 175 μm datasets. Dots represent the average F1 score for each model when processing the nine test videos. Lines represent the mean F1 scores averaged over different numbers of training frames. Shaded regions represent standard error. Horizontal lines are the average F1 scores of Suite2p and CaImAn. Download Figure 2-2, TIF file. [file eneuro-11-ENEURO.0352-23.2024-s009.tif]

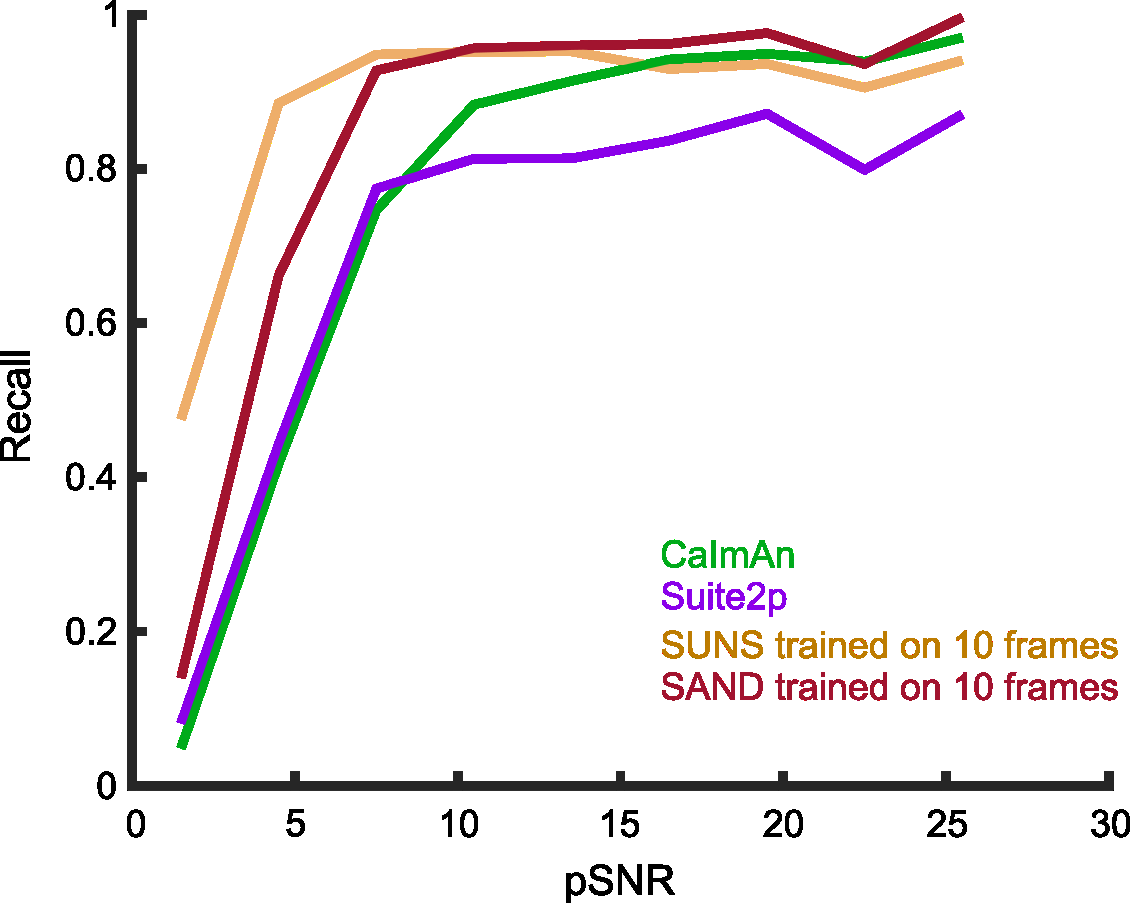

Supplement: Figure 2-3 — Neuron recall with SAND outperformed unsupervised algorithms. We calculated recall as a function of neuron pSNR for each method on the ABO 275 μm dataset. SAND trained on 10 frames could more reliability detect neurons, specifically in the low pSNR regime, compared to CaImAn and Suite2p. SUNS trained on 10 frames had higher recall than SAND in the low pSNR regime, but also had lower precision than all other methods (Figure 2C). Lines represent the average recall across all 10 models for all 10 videos. Neurons were grouped by their pSNR in bins with a width of 3 (n = 3016 neurons). Download Figure 2-3, TIF file. [file eneuro-11-ENEURO.0352-23.2024-s010.tif]

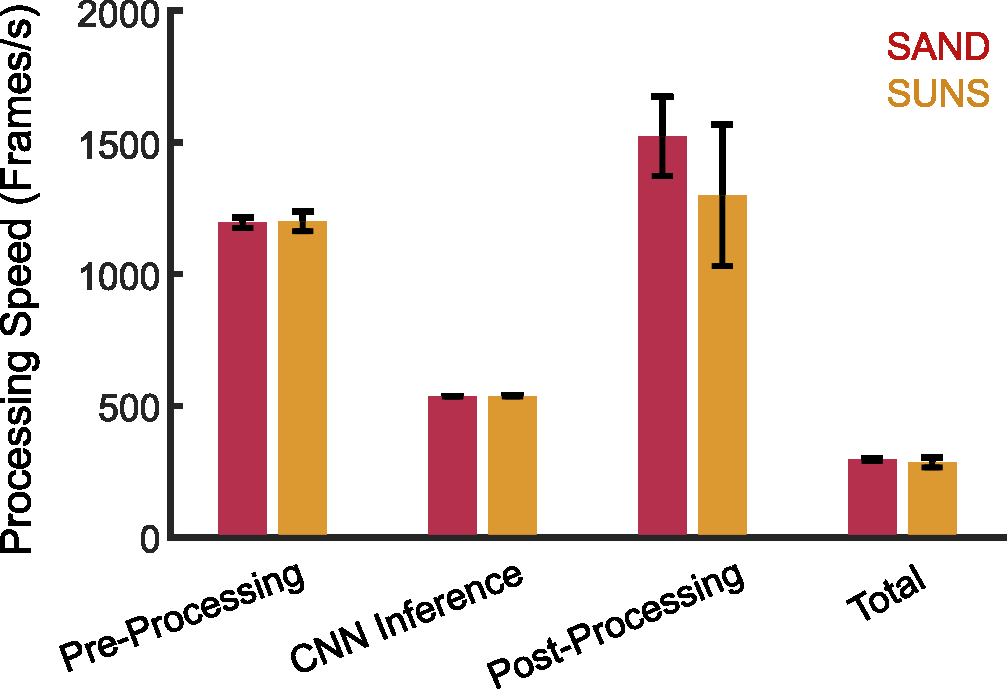

Supplement: Figure 2-4 — SAND achieved the same processing speed as SUNS on the ABO 275 μm dataset. Segmentation with SAND and SUNS consisted of three steps: pre-processing, CNN inference, and post-processing. For all steps, SAND and SUNS achieved comparable speeds. The total processing speed was an order of magnitude faster than the video’s frame rate (30 Hz). Bars represent the average processing speeds for 10 different models over 9 videos. Error bars represent the standard deviation. Download Figure 2-4, TIF file. [file eneuro-11-ENEURO.0352-23.2024-s011.tif]

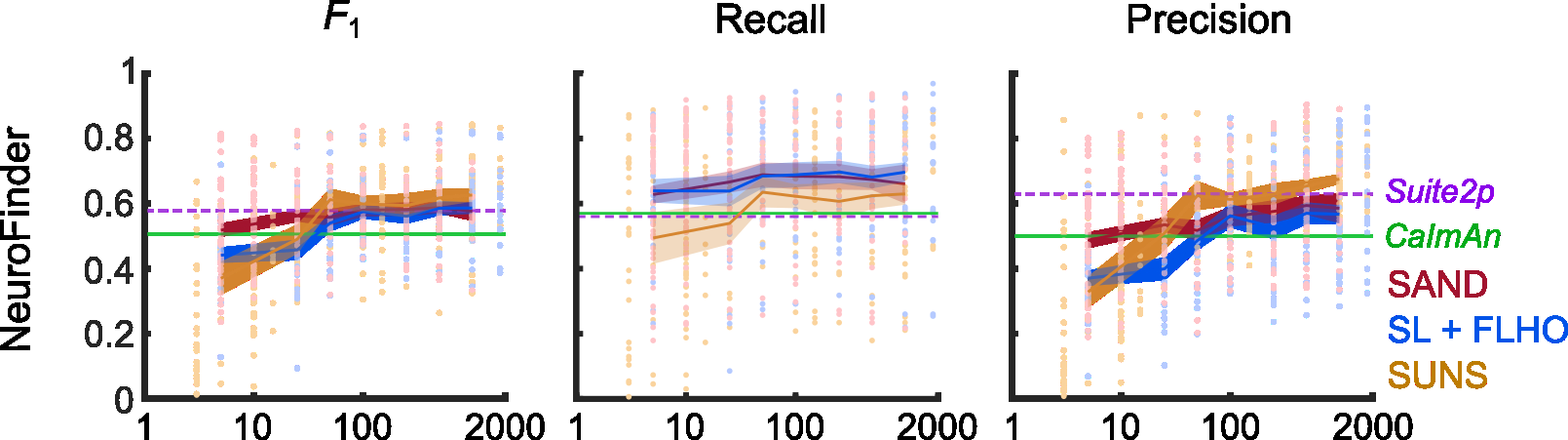

Supplement: Figure 3-1 — SAND outperforms SUNS on the Neurofinder dataset when trained on fewer labeled frames. SAND had higher precision and recall than SUNS when trained on only 10 labeled frames. Dots represent the average F1 score for each model when processing the nine test videos. Lines represent the mean F1 scores averaged over different numbers of training frames. Shaded regions represent standard error. Horizontal lines are the average F1 scores of Suite2p and CaImAn. Download Figure 3-1, TIF file. [file eneuro-11-ENEURO.0352-23.2024-s012.tif]

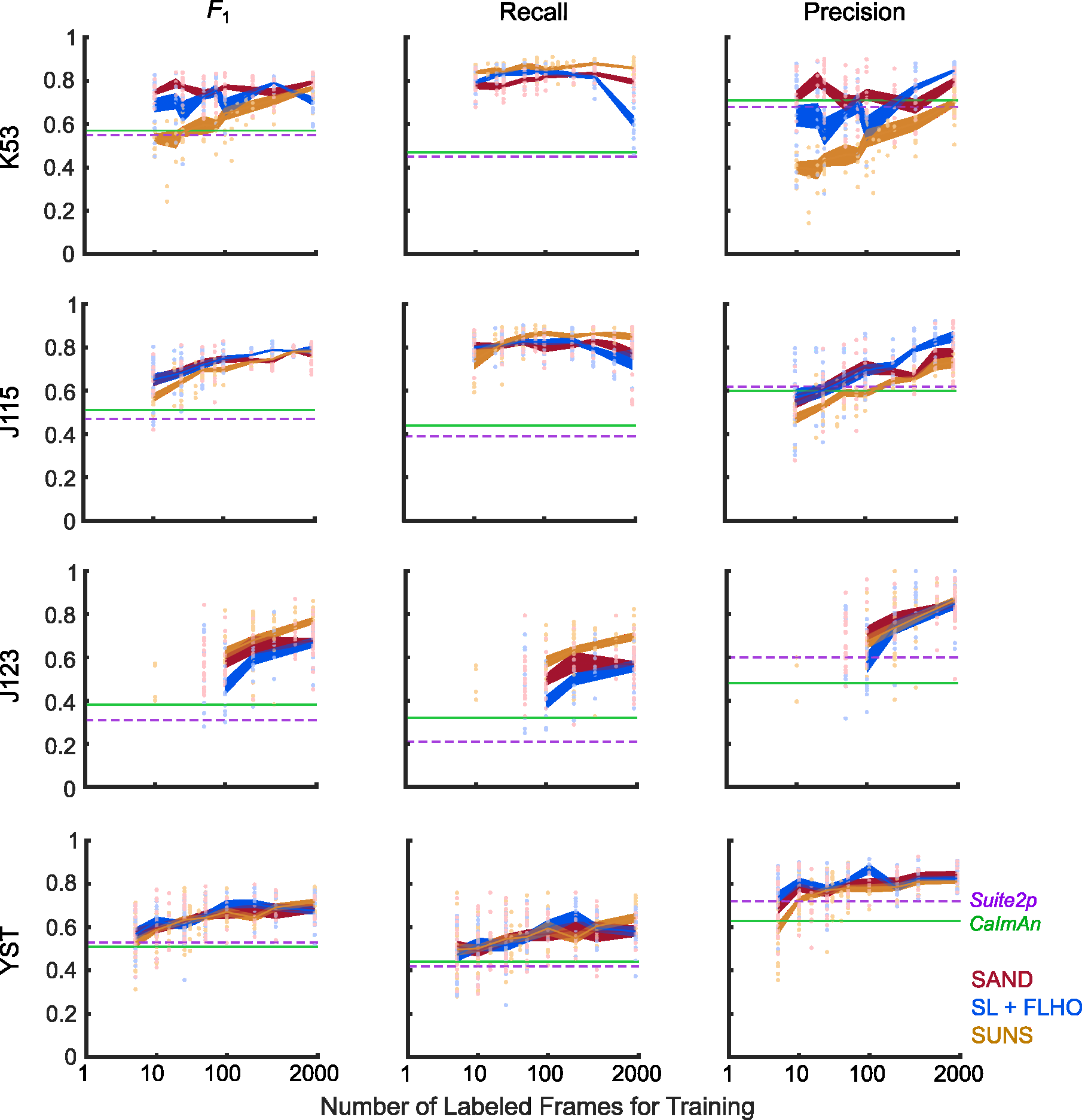

Supplement: Figure 4-1 — SAND outperforms SUNS on K53 and J115 when trained on fewer labeled frames. SAND had higher precision than SUNS when trained on only 10 labeled frames from the K53 and J115 videos. Dots represent the average F1 score for each model when processing the nine test videos. Lines represent the mean F1 scores averaged over different numbers of training frames. Shaded regions represent standard error. Horizontal lines are the average F1 scores of Suite2p and CaImAn. Download Figure 4-1, TIF file. [file eneuro-11-ENEURO.0352-23.2024-s013.tif]

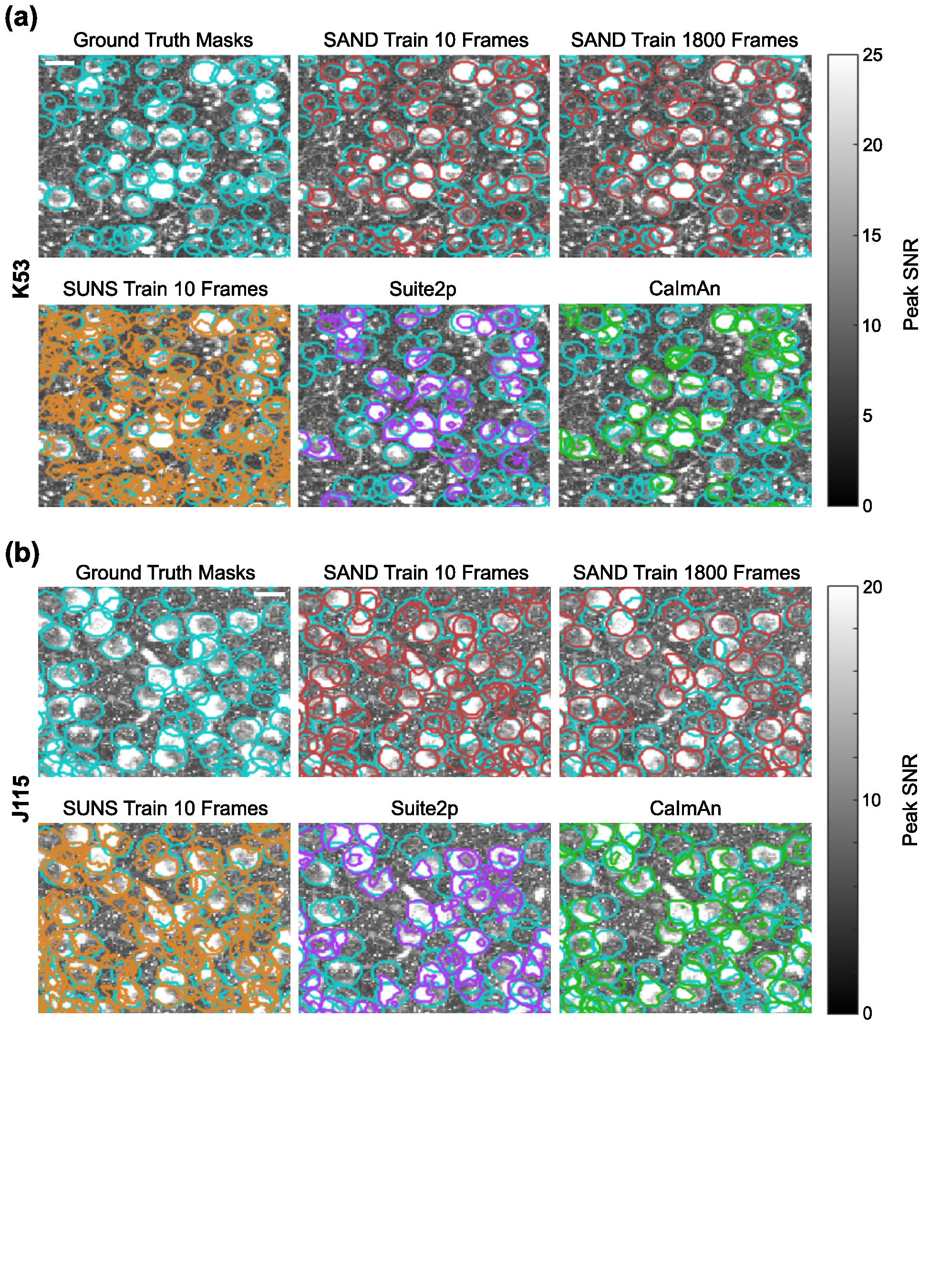

Supplement: Figure 4-2 — Masks generated by SAND closely matched ground truth masks on the K53 and J115 videos. (A) Example segmentations from a K53 sub-video. Masks generated by SAND were more accurate than those of other methods, even when trained on only 10 frames. The scale bar is 10 μm. (B) Example segmentations from a J115 sub-video. Masks generated by SAND were more accurate than those of other methods, even when trained on only 10 frames. For both videos, SUNS’s predictions included many false positives, while CaImAn and Suite2p had many false negatives. The scale bar is 10 μm. Download Figure 4-2, TIF file. [file eneuro-11-ENEURO.0352-23.2024-s014.tif]

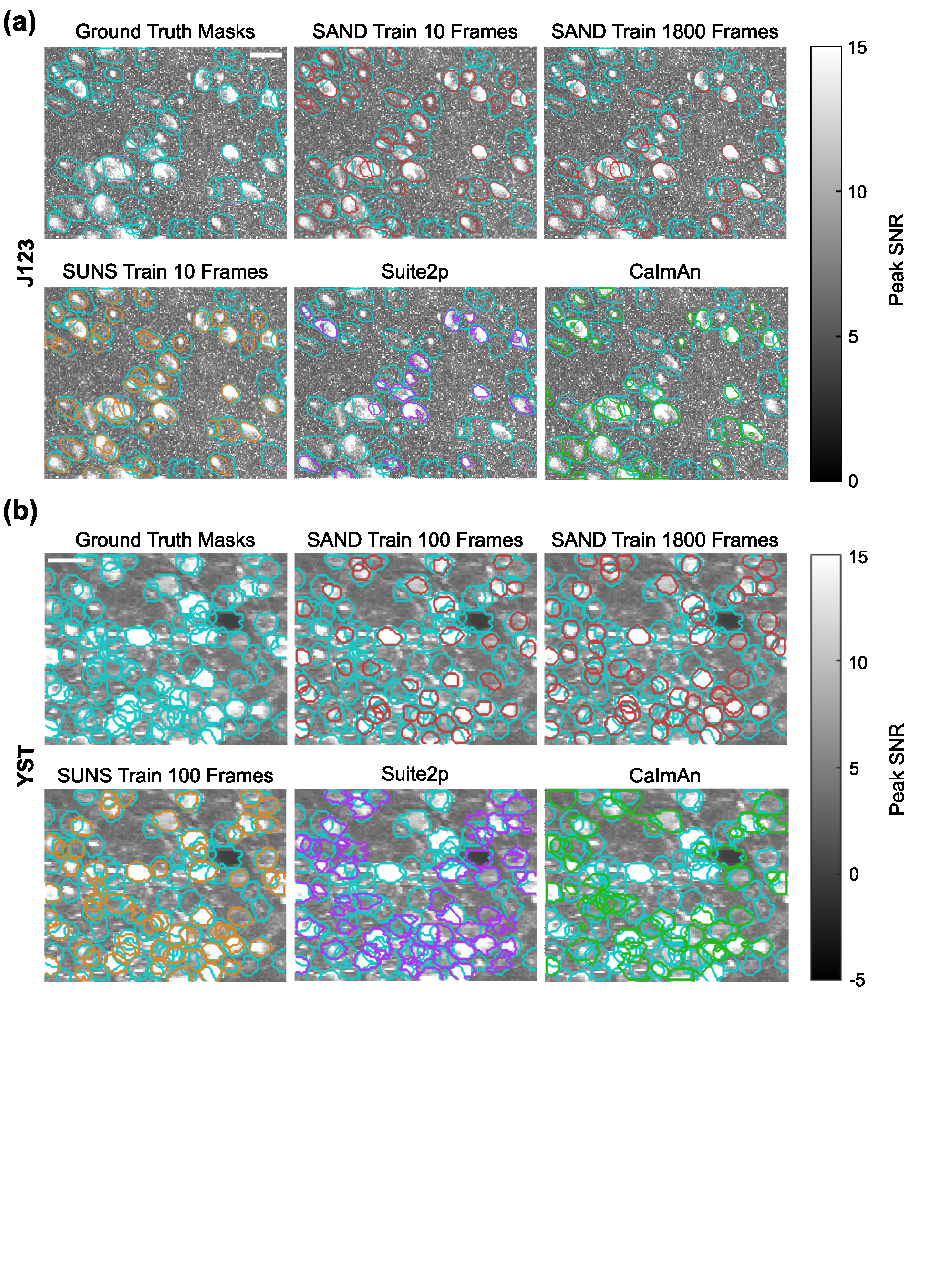

Supplement: Figure 4-3 — SAND and SUNS predicted similar neuron masks from the J123 and YST videos. (A) Example segmentations from a J123 sub-video. The scale bar is 25 μm. (B) Example segmentations from a YST sub-video. The scale bar is 10 μm. Download Figure 4-3, TIF file. [file eneuro-11-ENEURO.0352-23.2024-s015.tif]

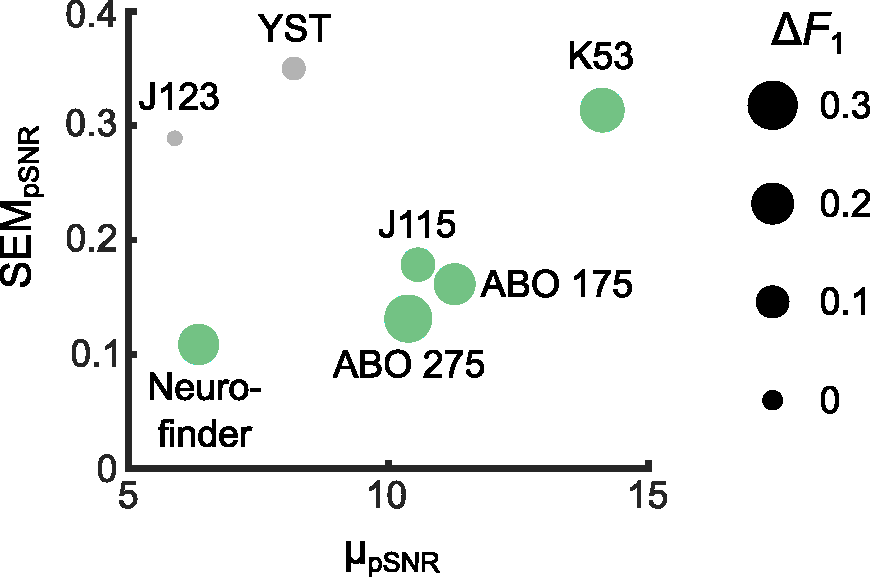

Supplement: Figure 4-4 — SAND greatly outperformed SUNS on datasets with high average pSNR or low variability of pSNR. Scatter plot of average vs standard error of pSNR for all neurons in each video. Dot size indicates the difference in median F1 between SAND and SUNS when trained on 0-50 neurons. Green dots indicate datasets where SAND significantly outperformed SUNS on low numbers of ground truth labels. Download Figure 4-4, TIF file. [file eneuro-11-ENEURO.0352-23.2024-s016.tif]

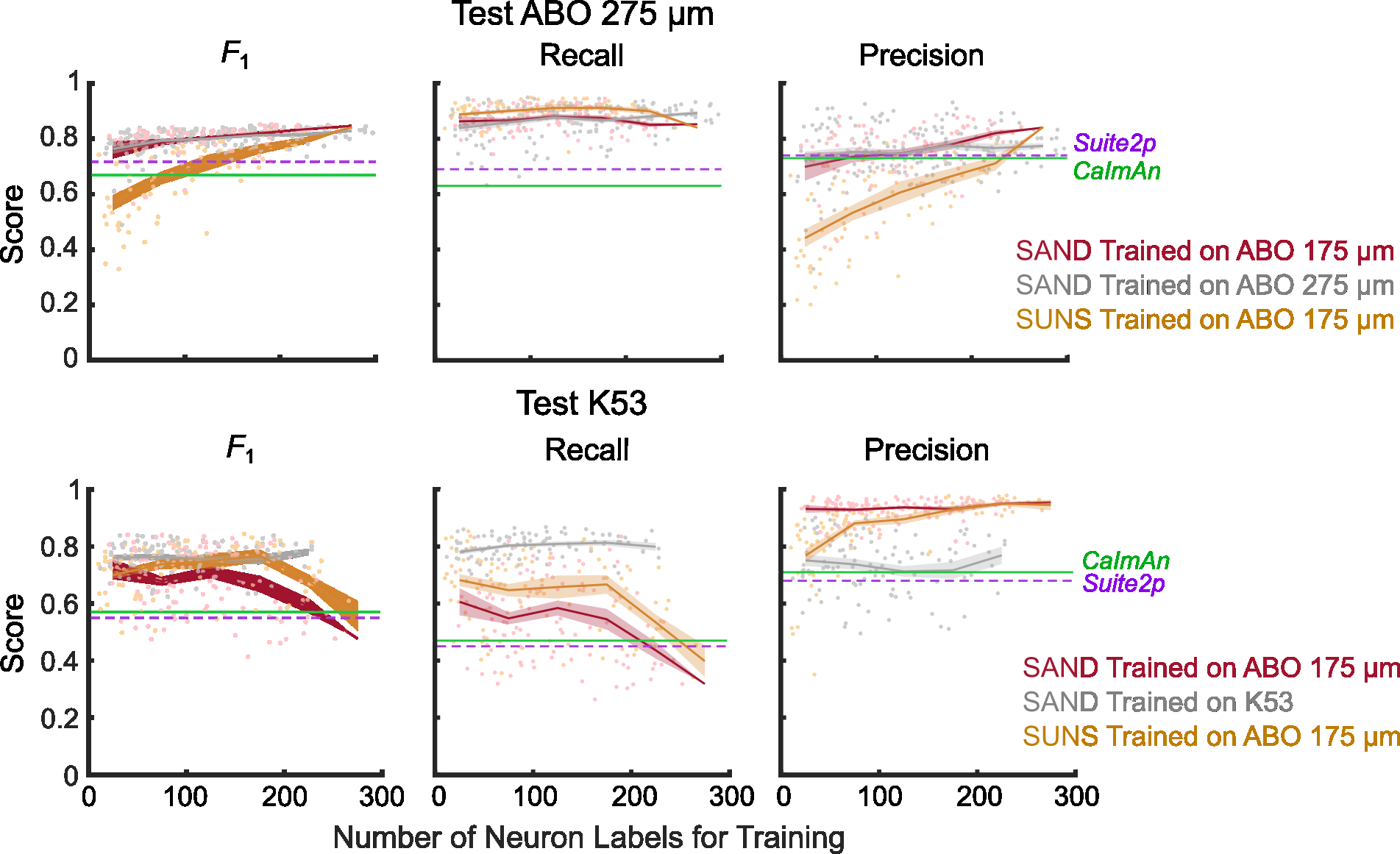

Supplement: Figure 4-5 — SAND generalized well to videos with similar imaging conditions as the training data. We trained SAND (red) and SUNS (orange) on the ABO 175 μm dataset and tested the performance of those models on a dataset with similar imaging conditions (ABO 275 μm) and a dataset with different imaging conditions (K53). We evaluated these models against CaImAn (green) and Suite2p (purple) using the optimal hyperparameters for each test dataset. We also evaluated the models against SAND models that were trained on videos with the same imaging conditions as the test data (gray). SAND generalized well to datasets with similar imaging conditions to the training data. SAND outperformed unsupervised methods when generalizing to data with different imaging conditions. However, SAND performed best when training and testing videos had the same imaging conditions. Download Figure 4-5, TIF file. [file eneuro-11-ENEURO.0352-23.2024-s017.tif]
