# Supplementary material for: A Semi-supervised Pipeline for Accurate Neuron Segmentation with Fewer Ground Truth Labels
Source: eNeuro. 2024 Feb 9;11(2):ENEURO.0352-23.2024. doi: 10.1523/ENEURO.0352-23.2024 (PMC10880440; doi:10.1523/ENEURO.0352-23.2024)
Supplement: Table 1-1 — The grid search values for post-processing hyperparameters were generally consistent between datasets. Values are listed as Begin:Step:End. Download Table 1-1, DOCX file. [file eneuro-11-ENEURO.0352-23.2024-s027.docx]

**Table 1-1:** **The grid search values for post-processing hyperparameters were generally consistent between datasets.** Values are listed as Begin:Step:End.

| Dataset | *p_thresh* | *min_area* (μm^2^) | *centroid_dist* (μm) | *min_consecutive* |
| --- | --- | --- | --- | --- |
| ABO | 0.64:0.02:0.8 | 54.8:3.04:82.1 | 3.1:0.8:6.2 | 1:1:7 |
| Neurofinder | 0.5:0.02:0.78 | 30.4:6.08:82.1 | 3.1:0.8:6.2 | 1:1:7 |
| K53 | 0.5:0.02:0.78 | 30.4:6.08:82.1 | 3.1:0.8:6.2 | 1:1:7 |
| J115 | 0.5:0.02:0.78 | 30.4:6.08:82.1 | 3.1:0.8:6.2 | 1:1:7 |
| J123 | 0.5:0.02:0.78 | 18.3:3.04:82.1 | 3.1:0.8:6.2 | 1:1:7 |
| YST | 0.5:0.02:0.78 | 18.3:3.04:82.1 | 3.1:0.8:6.2 | 1:1:7 |
