# Supplementary material for: A Semi-supervised Pipeline for Accurate Neuron Segmentation with Fewer Ground Truth Labels
Source: eNeuro. 2024 Feb 9;11(2):ENEURO.0352-23.2024. doi: 10.1523/ENEURO.0352-23.2024 (PMC10880440; doi:10.1523/ENEURO.0352-23.2024)
Supplement: Table 1-2 — The datasets in this study covered multiple brain regions and imaging conditions. V1 was primary visual cortex, PPC was posterior parietal cortex, S1 was primary somatosensory cortex, vS1 was vibrissal primary somatosensory cortex. Download Table 1-2, DOCX file. [file eneuro-11-ENEURO.0352-23.2024-s026.docx]

**Table 1-2**: **The datasets in this study covered multiple brain regions and imaging conditions.** V1 was primary visual cortex, PPC was posterior parietal cortex, S1 was primary somatosensory cortex, vS1 was vibrissal primary somatosensory cortex.

| Dataset | # Videos | Brain Region(s) | Sensor(s) | Frame Rate |
| --- | --- | --- | --- | --- |
| ABO | 20 | V1 | GCaMP6f | 30 Hz |
| Neurofinder | 12 | PPC, S1, V1, vS1 | GCaMP6s | 3 to 8 Hz |
| CaImAn | 4 | Hippocampus, PPC, V1 | GCaMP(3,5,6f) | 10 or 30 Hz |
