# Supplementary material for: A Semi-supervised Pipeline for Accurate Neuron Segmentation with Fewer Ground Truth Labels
Source: eNeuro. 2024 Feb 9;11(2):ENEURO.0352-23.2024. doi: 10.1523/ENEURO.0352-23.2024 (PMC10880440; doi:10.1523/ENEURO.0352-23.2024)
Supplement: Supplementary 1 — Download Suppl 1, ZIP file. [file eneuro-11-ENEURO.0352-23.2024-s018.zip › utils/create_h5_video_NF.m]

SUNS\_paper\_reproduction/create\_h5\_video\_NF.m at main · YijunBao/SUNS\_paper\_reproduction


Skip to content


- In this repository

  All GitHub
  ↵

  Jump to
  ↵

- No suggested jump to results

- In this repository

  All GitHub
  ↵

  Jump to
  ↵
- In this user

  All GitHub
  ↵

  Jump to
  ↵
- In this repository

  All GitHub
  ↵

  Jump to
  ↵

Dashboard

Pull requests
Issues
Codespaces

Marketplace

Explore
Sponsors
Settings


anonymous1neurips2023


Sign out

New repository

Import repository

New codespace

New gist

New organization

Sorry, something went wrong.

{{ message }}

/  ...  /

YijunBao  /  
SUNS\_paper\_reprod...  /  

Clear Command Palette


Tip:
Type `#` to search pull requests

Type `?` for help and tips


Tip:
Type `#` to search issues

Type `?` for help and tips


Tip:
Type `#` to search discussions

Type `?` for help and tips


Tip:
Type `!` to search projects

Type `?` for help and tips


Tip:
Type `@` to search teams

Type `?` for help and tips


Tip:
Type `@` to search people and organizations

Type `?` for help and tips


Tip:
Type `>` to activate command mode

Type `?` for help and tips


Tip:
Go to your accessibility settings to change your keyboard shortcuts

Type `?` for help and tips


Tip:
Type author:@me to search your content

Type `?` for help and tips


Tip:
Type is:pr to filter to pull requests

Type `?` for help and tips


Tip:
Type is:issue to filter to issues

Type `?` for help and tips


Tip:
Type is:project to filter to projects

Type `?` for help and tips


Tip:
Type is:open to filter to open content

Type `?` for help and tips


We’ve encountered an error and some results aren't available at this time. Type a new search or try again later.


No results matched your search


Search for **issues** and **pull requests**

`#`

Search for **issues, pull requests, discussions,** and **projects**

`#`

Search for **organizations, repositories,** and **users**

`@`

Search for **projects**

`!`

Search for **files**

`/`

Activate **command mode**

`>`

Search your issues, pull requests, and discussions

`# author:@me`

Search your issues, pull requests, and discussions

`# author:@me`

Filter to pull requests

`# is:pr`

Filter to issues

`# is:issue`

Filter to discussions

`# is:discussion`

Filter to projects

`# is:project`

Filter to open issues, pull requests, and discussions

`# is:open`

YijunBao
 
/
**SUNS\_paper\_reproduction**
Public

- Watch

  Couldn't load subscription status. 
   
  Retry
- Fork
  1
   Fork your own copy of YijunBao/SUNS\_paper\_reproduction
- ## Unstar this repository?

  This will remove {{ repoNameWithOwner }} from the {{ listsWithCount }} that it's been added to.

  Unstar

  Starred
   5

  Star
   5

- Code
- Issues
  0
- Pull requests
  0
- Discussions
- Actions
- Projects
  0
- Security
- Insights

More


- Code
- Issues
- Pull requests
- Discussions
- Actions
- Projects
- Security
- Insights

Open in github.dev
Open in a new github.dev tab
Open in codespace

Beta

Try the new code view

Permalink

main

Switch branches/tags


Branches
Tags

Could not load branches


Nothing to show

{{ refName }}
default
View all branches

Could not load tags


Nothing to show


{{ refName }}
default
View all tags

# Name already in use

A tag already exists with the provided branch name. Many Git commands accept both tag and branch names, so creating this branch may cause unexpected behavior. Are you sure you want to create this branch?

 Cancel
 Create

## SUNS\_paper\_reproduction/paper\_reproduction/utils/**create\_h5\_video\_NF.m**

 Go to file

 

- Go to file
  T
- Go to line
  L
- Copy path
- Copy permalink

This commit does not belong to any branch on this repository, and may belong to a fork outside of the repository.

Cannot retrieve contributors at this time

31 lines (26 sloc)
856 Bytes

Raw
 
Blame

Edit this file

E


Open in github.dev

.


Open in GitHub Desktop

- Open with Desktop
- View raw
- Copy raw contents
   Copy raw contents

   Copy raw contents

   Copy raw contents
- View blame
- Edit file
- Open with github.dev
- Delete file

This file contains bidirectional Unicode text that may be interpreted or compiled differently than what appears below. To review, open the file in an editor that reveals hidden Unicode characters.
Learn more about bidirectional Unicode characters

Show hidden characters


|  |  |
| --- | --- |
|  | % modified from |
|  | % 'https://github.com/soltanianzadeh/STNeuroNet/blob/master/demo\_preprocess.m' |
|  | % Released under a GPL v2 license. |
|  |  |
|  | %% |
|  | opt.type = 'test'; %{'train','test'} |
|  |  |
|  | name = {'100', '101', '200', '201', '400', '401'}; % |
|  | name\_long = {'01.00', '01.01', '02.00', '02.01', '04.00', '04.01'}; |
|  | DirData\_full = ['E:\NeuroFinder\',opt.type,' videos\']; % folder of the original videos |
|  | DirData = ['E:\NeuroFinder\',opt.type,' videos\']; % folder of the cropped videos |
|  |  |
|  | if strcmp(opt.type, 'train') |
|  | apd = ''; |
|  | elseif strcmp(opt.type, 'test') |
|  | apd = '.test'; |
|  | end |
|  |  |
|  | %% |
|  | for i = 1:length(name) |
|  | opt.ID = name{i}; |
|  | disp(opt.ID); |
|  | dataFile = fullfile(DirData,[name\_long{i},apd,'.h5']); |
|  | if ~exist(dataFile) |
|  | disp('Create cropped raw video'); |
|  | tic; |
|  | vid = prepareNeurofinder(opt, DirData\_full, DirData); |
|  | toc; |
|  | end |
|  | end |
|  |  |

- Copy lines
- Copy permalink
- View git blame
- Reference in new issue
- Reference in new discussion

 Go
 

Give feedback


# Provide feedback

## Footer

© 2023 GitHub, Inc.

### Footer navigation

- Terms
- Privacy
- Security
- Status
- Docs
- Contact GitHub
- Pricing
- API
- Training
- Blog
- About

You can’t perform that action at this time.

You signed in with another tab or window. Reload to refresh your session.
You signed out in another tab or window. Reload to refresh your session.
