# Supplementary material for: A Semi-supervised Pipeline for Accurate Neuron Segmentation with Fewer Ground Truth Labels
Source: eNeuro. 2024 Feb 9;11(2):ENEURO.0352-23.2024. doi: 10.1523/ENEURO.0352-23.2024 (PMC10880440; doi:10.1523/ENEURO.0352-23.2024)
Supplement: Table 2-2 — SAND had significantly higher quality masks than competing methods on the ABO datasets. We measured quality as the ratio of the mask’s area to the area of the mask's convex hull. SAND and SUNS used 10-fold cross validation to test performance; each model trained on a single video and was tested on the 9 remaining videos in that dataset (i.e. 9 applied models per video). CaImAn and Suite2p did not use cross validation (1 applied model per video). We evaluated SAND and SUNS based on models trained on 10 labeled frames. "# Training Frames" for Suite2p and CaImAn are N/A because these methods were unsupervised. We compared methods using a two-tailed Wilcoxon rank-sum test on all the masks generated by each model across all test videos. Download Table 2-2, DOCX file. [file eneuro-11-ENEURO.0352-23.2024-s024.docx]

**Table 2-2: SAND had significantly higher quality masks than competing methods on the ABO datasets.** We measured quality as the ratio of the mask’s area to the area of the mask’s convex hull. SAND and SUNS used 10-fold cross validation to test performance; each model trained on a single video and was tested on the 9 remaining videos in that dataset (i.e. 9 applied models per video). CaImAn and Suite2p did not use cross validation (1 applied model per video). We evaluated SAND and SUNS based on models trained on 10 labeled frames. “# Training Frames” for Suite2p and CaImAn are N/A because these methods were unsupervised. We compared methods using a two-tailed Wilcoxon rank-sum test on all the masks generated by each model across all test videos.

**ABO 275 μm**

| Method 1  (# Training Frames) | # predicted masks  (# applied models per video) | Method 2  (# Training Frames) | # predicted masks  (# applied models per video) | *p* |
| --- | --- | --- | --- | --- |
| SAND (10) | 30331 (9) | SUNS (10) | 70229 (9) | < 1.0 × 10^−320^ |
|  |  | Suite2p (N/A) | 2735 (1) | < 1.0 × 10^−320^ |
|  |  | CaImAn (N/A) | 2656 (1) | 5.2 × 10^−64^ |

**ABO 175 μm**

| Method 1  (# Training Frames) | # predicted masks  (# applied models per video) | Method 2  (# Training Frames) | # predicted masks  (# applied models per video) | *p* |
| --- | --- | --- | --- | --- |
| SAND (10) | 24114 (9) | SUNS (10) | 27938 (9) | < 1.0 × 10^−320^ |
|  |  | Suite2p (N/A) | 1992 (1) | < 1.0 × 10^−320^ |
|  |  | CaImAn (N/A) | 1863 (1) | 6.3 × 10^−41^ |
