# Supplementary material for: A Semi-supervised Pipeline for Accurate Neuron Segmentation with Fewer Ground Truth Labels
Source: eNeuro. 2024 Feb 9;11(2):ENEURO.0352-23.2024. doi: 10.1523/ENEURO.0352-23.2024 (PMC10880440; doi:10.1523/ENEURO.0352-23.2024)
Supplement: Table 2-3 — SAND had significantly higher quality masks than competing methods on the Neurofinder dataset. We measured quality as the ratio of the mask's area to the area of the mask’s convex hull. SAND and SUNS used Train 1 Test 1 cross validation to test performance; each model was trained on 1 video and tested on a corresponding video (i.e. 1 applied model per video). CaImAn and Suite2p did not use cross validation (1 applied model per video). We evaluated SAND and SUNS based on models trained on 10 labeled frames. "# Training Frames" for Suite2p and CaImAn are N/A because these methods were unsupervised. We compared methods using a two-tailed Wilcoxon rank-sum test on all the masks generated by each model across all test videos. Download Table 2-3, DOCX file. [file eneuro-11-ENEURO.0352-23.2024-s023.docx]

**Table 2-3: SAND had significantly higher quality masks than competing methods on the Neurofinder dataset.** We measured quality as the ratio of the mask’s area to the area of the mask’s convex hull. SAND and SUNS used Train 1 Test 1 cross validation to test performance; each model was trained on 1 video and tested on a corresponding video (i.e. 1 applied model per video). CaImAn and Suite2p did not use cross validation (1 applied model per video). We evaluated SAND and SUNS based on models trained on 10 labeled frames. “# Training Frames” for Suite2p and CaImAn are N/A because these methods were unsupervised. We compared methods using a two-tailed Wilcoxon rank-sum test on all the masks generated by each model across all test videos.

**Neurofinder**

| Method 1  (# Training Frames) | # predicted masks  (# applied models per video) | Method 2  (# Training Frames) | # predicted masks  (# applied models per video) | *p* |
| --- | --- | --- | --- | --- |
| SAND (10) | 3909 (1) | SUNS (10) | 4185 (1) | < 1.0 × 10^−320^ |
|  |  | Suite2p (N/A) | 2916 (1) | < 1.0 × 10^−320^ |
|  |  | CaImAn (N/A) | 3192 (1) | 1.4 × 10^−106^ |
