# Supplementary material for: A Semi-supervised Pipeline for Accurate Neuron Segmentation with Fewer Ground Truth Labels
Source: eNeuro. 2024 Feb 9;11(2):ENEURO.0352-23.2024. doi: 10.1523/ENEURO.0352-23.2024 (PMC10880440; doi:10.1523/ENEURO.0352-23.2024)
Supplement: Table 2-4 — SAND had significantly higher quality masks than competing methods on the CaImAn datasets. We measured quality as the ratio of the mask's area to the area of the mask's convex hull. SAND and SUNS trained on 1 video for the K53 and J115 datasets and tested on the remaining 3 videos (i.e. 3 applied models per video). SAND and SUNS trained on 3 videos for the J123 and YST datasets and tested on the held-out video (1 applied model per video). "# Training Frames" for Suite2p and CaImAn are N/A because these methods were unsupervised. We compared methods using a two-tailed Wilcoxon rank-sum test on all the masks generated by each model across all test videos. Download Table 2-4, DOCX file. [file eneuro-11-ENEURO.0352-23.2024-s022.docx]

**Table 2-4: SAND had significantly higher quality masks than competing methods on the CaImAn datasets.** We measured quality as the ratio of the mask’s area to the area of the mask’s convex hull. SAND and SUNS trained on 1 video for the K53 and J115 datasets and tested on the remaining 3 videos (i.e. 3 applied models per video). SAND and SUNS trained on 3 videos for the J123 and YST datasets and tested on the held-out video (1 applied model per video). “# Training Frames” for Suite2p and CaImAn are N/A because these methods were unsupervised. We compared methods using a two-tailed Wilcoxon rank-sum test on all the masks generated by each model across all test videos.

**K53**

| Method 1  (# Training Frames) | # predicted masks  (# applied models per video) | Method 2  (# Training Frames) | # predicted masks  (# applied models per video) | *p* |
| --- | --- | --- | --- | --- |
| SAND (10) | 2842 (3) | SUNS (10) | 4945 (3) | < 1.0 × 10^−320^ |
|  |  | Suite2p (N/A) | 575 (1) | 1.1 × 10^−182^ |
|  |  | CaImAn (N/A) | 582 (1) | 1.5 × 10^−83^ |

**J115**

| Method 1  (# Training Frames) | # predicted masks  (# applied models per video) | Method 2  (# Training Frames) | # predicted masks  (# applied models per video) | *p* |
| --- | --- | --- | --- | --- |
| SAND (10) | 3630 (3) | SUNS (10) | 4307 (3) | < 1.0 × 10^−320^ |
|  |  | Suite2p (N/A) | 524 (1) | 4.9 × 10^−195^ |
|  |  | CaImAn (N/A) | 605 (1) | 1.9 × 10^−82^ |

**J123**

| Method 1  (# Training Frames) | # predicted masks  (# applied models per video) | Method 2  (# Training Frames) | # predicted masks  (# applied models per video) | *p* |
| --- | --- | --- | --- | --- |
| SAND (100) | 105 (1) | SUNS (100) | 134 (1) | 7.6 × 10^−5^ |
|  |  | Suite2p (N/A) | 55 (1) | 1.4 × 10^−15^ |
|  |  | CaImAn (N/A) | 102 (1) | 1.3 × 10^−5^ |

**YST**

| Method 1  (# Training Frames) | # predicted masks  (# applied models per video) | Method 2  (# Training Frames) | # predicted masks  (# applied models per video) | *p* |
| --- | --- | --- | --- | --- |
| SAND (10) | 207 (1) | SUNS (10) | 207 (1) | 9.5 × 10^−11^ |
|  |  | Suite2p (N/A) | 201 (1) | 1.0 × 10^−50^ |
|  |  | CaImAn (N/A) | 235 (1) | 7.7 × 10^−10^ |
