# Supplementary material for: A Semi-supervised Pipeline for Accurate Neuron Segmentation with Fewer Ground Truth Labels
Source: eNeuro. 2024 Feb 9;11(2):ENEURO.0352-23.2024. doi: 10.1523/ENEURO.0352-23.2024 (PMC10880440; doi:10.1523/ENEURO.0352-23.2024)
Supplement: Table 3-1 — SAND significantly outperformed SUNS on the Neurofinder dataset when both trained on a small number of labels. SAND trained on 0-50 labels did not significantly differ from SUNS trained on 200-250 labels. "Neuron #" indicates the range of labeled neurons used to train our models over many trials, grouped as a bin; the average number of training labels for each of these trials followed in parentheses. "Neuron #" for CaImAn and Suite2p is listed as NA because these methods were unsupervised; however, hyperparameter optimization for these unsupervised methods was done using all ground truth labels. nx shows the number of trials (models) in that bin. F1 is the median F1 value in that bin. We used a two-sided Wilcoxon rank-sum test to evaluate significance. *, **, ***, and n.s. represent p < 0.05, 0.01, 0.001, and not significant, respectively. Download Table 3-1, DOCX file. [file eneuro-11-ENEURO.0352-23.2024-s021.docx]

**Table 3-1: SAND significantly outperformed SUNS on the Neurofinder dataset when both trained on a small number of labels**. SAND trained on 0-50 labels did not significantly differ from SUNS trained on 200-250 labels. “Neuron #” indicates the range of labeled neurons used to train our models over many trials, grouped as a bin; the average number of training labels for each of these trials followed in parentheses. “Neuron #” for CaImAn and Suite2p is listed as NA because these methods were unsupervised; however, hyperparameter optimization for these unsupervised methods was done using all ground truth labels. *n_x_* shows the number of trials (models) in that bin. *F*_1_ is the median *F*_1_ value in that bin. We used a two-sided Wilcoxon rank-sum test to evaluate significance. *, **, ***, and n.s. represent *p* < 0.05, 0.01, 0.001, and not significant, respectively.

| Method 1 | Neuron #  (mean) | *n*_1_ | *F*_1_ | Method 2 | Neuron #  (mean) | *n*_2_ | *F*_1_ | *p* | *d* |
| --- | --- | --- | --- | --- | --- | --- | --- | --- | --- |
| SAND | 0-50  (32) | 43 | **0.53** | SUNS | 0-50  (29) | 43 | 0.34 | 1.4 × 10^-4^  (***) | 1.05 |
| SAND | 0-50  (32) | 43 | **0.53** | SL+FLHO | 0-50  (31) | 13 | 0.44 | 0.068  (n.s.) | 0.62 |
| SAND | 0-50  (32) | 43 | **0.53** | CaImAn | NA | 12 | 0.49 | 0.80  (n.s.) | 0.02 |
| SAND | 0-50  (32) | 43 | 0.53 | Suite2p | NA | 12 | **0.60** | 0.03  (*) | 0.57 |
| SAND | 0-50  (32) | 43 | 0.53 | SUNS | 200-250 (231) | 46 | **0.59** | 0.064  (n.s.) | 0.34 |
| SAND | 50-100 (76) | 57 | 0.55 | SUNS | 200-250 (231) | 46 | **0.59** | 0.77  (n.s.) | 0.01 |
| SAND | 200-250 (227) | 37 | 0.56 | SUNS | 200-250 (231) | 46 | **0.59** | 0.89  (n.s.) | 0.09 |
