# Supplementary material for: A Semi-supervised Pipeline for Accurate Neuron Segmentation with Fewer Ground Truth Labels
Source: eNeuro. 2024 Feb 9;11(2):ENEURO.0352-23.2024. doi: 10.1523/ENEURO.0352-23.2024 (PMC10880440; doi:10.1523/ENEURO.0352-23.2024)
Supplement: Table 4-2 — Calcium sensor SNR have improved over multiple generations of development. SNR values are the average fold increase of SNR relative to GCaMP3. Values are estimated from (Y. Zhang et al., 2023) and (Chen et al., 2013) and are based on single action potential results in vitro. Download Table 4-2, DOCX file. [file eneuro-11-ENEURO.0352-23.2024-s019.docx]

**Table 4-2: Calcium sensor SNR have improved over multiple generations of development.** SNR values are the average fold increase of SNR relative to GCaMP3. Values are estimated from (Y. Zhang et al., 2023) and (Chen et al., 2013) and are based on single action potential results *in vitro*.

| GCaMP | 3 | 5G | 6f | 6s | 7f | 8f | 7s | 8s |
| --- | --- | --- | --- | --- | --- | --- | --- | --- |
| SNR | 1 | 1.7 | 6.3 | 11.2 | 18.7 | 36.5 | 66.4 | 166.5 |
